# Advancing Wearable Technologies with Hydrogels: Innovations and Future Perspectives

**DOI:** 10.3390/gels11120988

**Published:** 2025-12-08

**Authors:** Kindness A. Uyanga, Ejike J. Onyeukwu, Jie Han

**Affiliations:** 1 School of Science and Technology, Hong Kong Metropolitan University, 81 Chung Hau Street, Kowloon, Hong Kong; onyeukwu@hkmu.edu.hk; 2 Department of Mechanical Engineering, City University of Hong Kong, 83 Tat Chee Avenue, Kowloon, Hong Kong

**Keywords:** smart hydrogels, hydrogel synthesis for wearable devices, conductive hydrogels, composite hydrogels, natural polymer hydrogels, wearable technology

## Abstract

Functionalized hydrogels represent an emerging class of smart materials being explored for advancing next-generation wearable technologies, owing to their flexibility, biocompatibility, stimuli-responsiveness, and tunable properties. This review provides an overview of recent developments in hydrogel-based wearables, highlighting their potential to enhance adaptive, multifunctional, and environmentally sustainable devices and textiles. It begins by examining progress in wearable sensors, energy storage and harvesting, biosignal monitoring, and smart textiles, as well as the associated challenges, including limited battery life, inadequate skin adhesion, user discomfort, and constrained functionality. The review further explores the synthesis, fabrication techniques, properties, and types of hydrogels tailored for wearable technologies, followed by a detailed discussion of their applications in smart batteries, supercapacitors, sensors, nanogenerators, fabrics and hybrid systems. It also highlights integrating artificial intelligence (AI) and the Internet of Things (IoT) to improve designs; enhance performance through real-time monitoring, data analytics, and user interaction; and expand functionality. Also, it analyzes key limitations of current hydrogels—particularly in energy density, dehydration resistance, fatigue behaviour, and large-scale reproducibility—and outlines strategies based on hierarchical material design, sustainable and biodegradable formulations, and standardized testing and regulatory alignment. The review concludes by affirming the role of hydrogel-based technologies in shaping the future of wearable innovations across healthcare, lifestyle, and beyond and outlines promising research directions.

## 1. Introduction

Wearable technology—smart devices and textiles worn on or integrated into the human body that combine materials, information, and electronics to collect, process and transmit feedback on biometric data [1]—has evolved rapidly since the 1960s from simple fitness trackers to sophisticated real-time health-monitoring systems. However, traditional wearables face limitations due to mechanical rigidity and poor biocompatibility of materials such as metals and rigid polymers, which restrict body movement and physiological monitoring. Flexible wearables made from hydrogels—soft, pliable, polymer-based structures that retain fluid while maintaining structural integrity [2,3,4]—offer a promising alternative, particularly as advances in artificial intelligence (AI) and the Internet of Things (IoT) continue.

Hydrogels possess several advantages for wearable applications: their high water content mimics natural tissues, enhancing biocompatibility and enabling long-term wear [5,6,7]; their inherent flexibility and stretchability conform to body movements, improving comfort over rigid materials; and their natural adhesiveness [2] eliminates the need for external adhesives, reducing allergic reactions. These properties address critical limitations in comfort, fit, and skin compatibility, positioning hydrogels as transformative materials for wearable systems.

**Figure 1:**
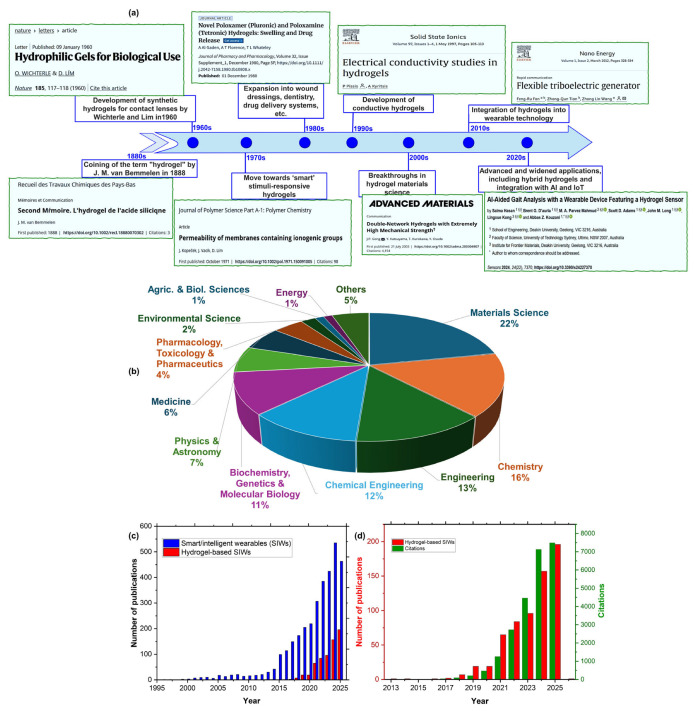
(**a**) Timeline of hydrogel development for wearables. (**b**) Research fields investigating hydrogels, with “Others” encompassing Computer Science, Immunology, Microbiology, Neuroscience, Health Professions, Mathematics, Earth Sciences, Dentistry, Nursing, Veterinary Science, and Psychology. (**c**) Publication trends in smart/intelligent wearables (SIWs) and hydrogel-based SIWs. (**d**) Publication volume and citations for hydrogel-based SIWs. The literature volumes for Figure 1 were obtained using the search queries (a) ((TITLE-ABS-KEY(hydrogel*) AND PUBYEAR > 1958 AND PUBYEAR < 2026) AND (hydrogel)), (c) “(TITLE-ABS-KEY(smart intelligent wearable*) AND PUBYEAR > 1995 AND PUBYEAR < 2027)”, and (d) “((TITLE-ABS-KEY(smart intelligent wearable*) AND PUBYEAR > 2012 AND PUBYEAR < 2027) AND (hydrogel*))” utilizing SCOPUS accessed 1 September 2025.

The term “hydrogel” was first coined around 1888 by researchers such as J.M. van Bemmelen to describe colloidal gels based on organic salts [8]. Modern hydrogels have been synthesized for over 70 years (Figure 1a), beginning in the 1960s with the development of poly(2-hydroxyethyl methacrylate) (pHEMA) for contact lenses by Wichterle and Lim [6,9]. The 1970s marked a pivotal shift toward stimuli-responsive “smart” hydrogels that respond to environmental factors such as temperature and pH [10,11,12,13,14]. The 1980s expanded applications to drug delivery, wound dressing, and dentistry [15,16], while the 1990s introduced electrically conductive hydrogels, enabling integration with electronics and biosensors [17]. Significant breakthroughs in the 2000s included stretchable, tough hydrogels and enhanced conductivity through carbon-based nanomaterials and metal nanoparticles [18,19]. Since 2010, hydrogel-based wearables have accelerated, with Wang et al.’s 2012 [20] introduction of triboelectric nanogenerators enabling energy harvesting. Recent research demonstrates applications in self-cooling fabrics, energy harvesting, self-powered sensing [21,22,23,24], and AI/IoT-integrated systems [25,26].

Hydrogels are increasingly explored (Figure 1b) for flexible wearable devices and textiles due to their biocompatibility, moisture absorption, and permeability. Their biodegradability—unlike traditional wearables—reduces e-waste and environmental pollution while supporting sustainable design. Their thermal responsiveness enables smart textiles, such as temperature-regulating garments. Growing publication and citation volumes since the 2010s reflect surging interest in hydrogel-based wearables and their potential to transform wearable technology through sustainable, multifunctional innovation (Figure 1c,d).

Despite the growing body of literature on hydrogel-based wearable technologies (Figure 1c,d), several gaps remain. Existing reviews are largely application-specific [27,28,29], focusing on areas such as bioelectronics [30], human–machine interfaces [5], batteries [2,31] and triboelectric nanogenerators [32], rather than offering a comprehensive, interdisciplinary perspective. Even the most recent broad review [33] concentrates on multifunctional hydrogels for wearable electronics, emphasizing electronic performance, material innovations, and device miniaturization, while paying insufficient attention to textile integration, user comfort, environmental sustainability, and the emerging role of AI and IoT. Furthermore, advanced fabrication techniques—such as 3D/4D printing, electrospinning, and microfabrication—essential for customized, skin-conforming designs are rarely addressed. A holistic approach that integrates materials science, device engineering, user experience, and sustainability is urgently needed but is currently absent from the literature.

This review fills these gaps by providing a truly interdisciplinary analysis. Unlike previous reviews that prioritize electronic performance alone, we place equal emphasis on user comfort, biocompatibility, and environmental sustainability—factors critical for long-term wearability and real-world adoption. Our work uniquely bridges hydrogel applications across both wearable devices and textiles, demonstrating seamless integration into everyday garments. It systematically examines advanced fabrication techniques enabling customizable and eco-friendly designs and explores the largely uncharted intersection of hydrogels with AI and IoT technologies. Specifically, this review highlights critical challenges in wearable devices and textiles, including performance metrics, stability, reproducibility, and target specifications. It compares conventional hydrogel performance against these target values, identifies gaps and limitations, and proposes strategies to overcome these challenges, ensuring next-generation wearables meet durability, comfort, and sustainability requirements. In addition, we comprehensively survey advancements since 2016 in hydrogel-based smart wearables—including sensors, energy storage and harvesting devices, smart fabrics, hybrid systems, and AI/IoT-enabled wearables—while analyzing unresolved issues such as mechanical durability and long-term stability. By integrating materials science, device engineering, health applications, and user-centered design, this review offers the most complete picture of hydrogel-based wearable technology to date and outlines strategic directions for sustainable, connected, and user-friendly innovations for next-generation wearables.

## 2. Overview of the State-of-the-Art Progress in Wearable Technologies

In recent years, wearable technologies have advanced rapidly, driven by innovations in flexible electronics, nanomaterials, and autonomous power systems, enabling continuous, real-time health monitoring through increasingly unobtrusive, multifunctional, and self-sustaining devices. These devices include activity trackers, augmented reality devices, microfluidic patches, smart fabrics, electrocardiogram and electroencephalogram devices, continuous vital sign collection (CVSC) devices, real-time glucose monitoring devices, smart rings, necklaces, contact lenses, and gloves [34]. Notable progress has been made in wearable sensors, energy storage, energy harvesting, biosignal monitoring, and smart textiles, though critical challenges remain in performance metrics, stability, and reproducibility (Table 1).

### 2.1. Wearable Sensors: Performance and Limitations

Recent progress in wearable sensors has focused on enhancing sensitivity, flexibility, stretchability, multifunctionality, and non-invasive monitoring, with emerging integration of AI and human–machine learning [35,36]. However, critical evaluation reveals significant variability in performance metrics and stability across different material platforms.

Advances in materials science have led to sensors with hybrid materials and structures capable of simultaneously measuring multiple parameters [37,38,39,40,41]. Graphene–metal nanoparticle hybrids represent a particularly promising approach. Lee et al. [41] demonstrated that doping graphene with metal nanoparticles and combining it with a gold mesh enhances electrochemical activity through synergistic effects: gold nanoparticles provide catalytic sites that improve electron transfer (achieving sensitivity of 10.93 μA/mM/cm^2^ for glucose detection), while graphene increases active surface area and electron transfer kinetics. This hybrid structure achieved conductivity of approximately 10^3^–10^4^ S/m and demonstrated stable performance over 100 bending cycles, making it suitable for sweat-based diabetes monitoring. However, the long-term stability beyond several weeks and reproducibility across manufacturing batches remain inadequately characterized.

Yu et al. [40] advanced this concept with a fully perspiration-powered electronic skin (PPES) featuring multimodal sensors and lactate biofuel cells utilizing zero- to three-dimensional nanomaterials. The system achieved remarkable power density (3.5 mW/cm^2^) and extended operational stability (60 h continuous operation), monitoring urea, glucose, pH, and NH_4_^+^, and skin temperature while wirelessly transmitting data via Bluetooth. Despite these achievements, critical limitations include: (1) performance degradation under varying environmental conditions (humidity, temperature fluctuations); (2) calibration drift requiring frequent recalibration; and (3) limited mechanistic understanding of ion vs. electron conductivity contributions to overall device performance. The device primarily relies on ion conductivity through sweat (typically 0.01–0.1 S/m), while the nanomaterial electrodes provide electron conductivity (1000–10,000 S/m). However, the interface between these conduction mechanisms remains a bottleneck affecting long-term reliability.

### 2.2. Energy Storage: Performance and Limitations

Energy storage remains a critical challenge for wearable systems, with key progress in flexible batteries and supercapacitors, yet current performance falls short of target values for extended autonomous operation.

Flexible lithium-ion and solid-state batteries offer mechanical flexibility while addressing safety concerns [42,43,44]. Current flexible lithium-ion batteries achieve energy densities of 100–200 Wh/kg—significantly lower than those of rigid counterparts (250–300 Wh/kg)—and exhibit capacity retention of 70–80% after 500 cycles of repeated bending (bending radius > 5 mm). Solid-state batteries improve safety but suffer from lower ionic conductivity (0.001–0.0001 S/cm at room temperature vs. 0.01–0.1 S/cm for liquid electrolytes), limiting power delivery. Target values for practical wearable applications include energy density >250 Wh/kg, capacity retention >90% after 1000 cycles under mechanical deformation, and operational temperature range of −20 °C to 60 °C—goals not yet achieved by current flexible battery technologies.

Supercapacitors utilizing carbon nanotube yarns and conductive polymers show promise for textile integration [45,46,47,48]. Carbon nanotubes provide electrical conductivity (10^4^–10^5^ S/m), high specific surface area (>1000 m^2^/g), and mechanical strength, while conductive polymers (e.g., PEDOT:PSS, polyaniline) enhance energy density (20–40 Wh/kg) through pseudocapacitance. Textile-integrated supercapacitors achieve power densities of 1–10 kW/kg but suffer from limited energy density (<10 Wh/kg vs. target >50 Wh/kg) and stability issues: capacitance retention typically degrades to 70–85% after 5000 cycles, and washing durability remains problematic (>30% performance loss after 10 wash cycles). Furthermore, reproducibility across different textile substrates and manufacturing processes shows high variability (±15–25% in capacitance values).

Hybrid energy storage systems combining batteries and supercapacitors address transient power demands while extending battery lifespan by 20–40% [49,50]. However, system complexity, increased weight (20–30% increase), and integration challenges limit practical deployment. Critical assessment reveals that current hybrid systems achieve only 60–70% of theoretical performance due to impedance mismatches and control inefficiencies.

### 2.3. Energy Harvesting: Performance and Limitations

Energy harvesting technologies convert ambient energy into electrical power, yet current power outputs remain insufficient for many wearable applications that require >1 mW of continuous power.

Triboelectric and piezoelectric nanogenerators harness mechanical energy from body movements [51,52,53], typically generating 10–500 μW/cm^2^ under normal walking conditions—adequate for ultra-low-power sensors but insufficient for wireless data transmission (which typically requires 1–10 mW). Thermoelectric generators utilizing body heat gradients face inherent limitations due to small temperature differences (typically 2–5 °C between skin and ambient) [54], with Vashaee’s team achieving notable performance: 44 μW/cm^2^ under still air and 156.5 μW/cm^2^ under airflow using nanocomposite bismuth telluride materials [55]. This represents 4–7× improvement over commercial TEGs, yet falls short of target values (>500 μW/cm^2^) for continuous operation of multifunctional wearables. Critical limitations include: (1) dependence on environmental conditions (ambient temperature, airflow); (2) thermoelectric figure of merit (ZT) limited to 0.8–1.2 for flexible materials vs. >2.0 for optimal performance; and (3) thermal contact resistance degradation over time (10–20% performance loss after extended wear).

Biofuel cells generating electricity from sweat or blood offer attractive power densities (1–5 mW/cm^2^ from sweat lactate) [56,57], as demonstrated by Caltech’s hybrid e-skin [58]. However, critical challenges include: (1) enzyme stability (50–70% activity loss within 1–2 weeks); (2) dependence on metabolite availability (requiring physical activity for sweat production); (3) biocompatibility concerns for implantable variants; and (4) reproducibility issues stemming from batch-to-batch enzyme variability (±20–30%).

### 2.4. Biosignal Monitoring and Smart Textiles: Performance and Limitations

Continuous biosignal monitoring (electrocardiography (ECG), electromyography (EMG), electroencephalography (EEG), and photoplethysmography (PPG)) has advanced with dry electrodes and tattoo-like sensors, yet signal quality and long-term stability remain critical concerns. Dry electrodes achieve skin-electrode impedance of 10–100 kΩ (vs. 1–10 kΩ for wet electrodes), resulting in signal-to-noise ratios 5–10 dB lower than those of clinical-grade systems [59,60,61]. AI-enhanced signal processing partially compensates, but motion artifacts and electrode drift necessitate frequent recalibration (every 2–4 h during continuous monitoring). Target impedance values < 10 kΩ and stability > 24 h without recalibration remain unmet.

Smart textiles integrating conductive yarns, embroidered circuits, and printed electronics [62,63,64,65] enable seamless body integration. Conductive yarns achieve resistivity of 0.01–0.1 Ω·cm (silver-coated) to 10–100 Ω·cm (carbon-based), but washing durability poses challenges: conductivity typically degrades 20–50% after 10–20 wash cycles. E-textiles incorporating energy storage, sensing, and communication demonstrate multifunctionality but suffer from: (1) limited mechanical durability (failure after 500–1000 stretch cycles at 30% strain); (2) breathability reduction (30–50% decrease in moisture vapour transmission rate); and (3) manufacturing complexity limiting scalability and reproducibility.

### 2.5. Critical Challenges and the Need for Hydrogel Integration

Despite impressive progress, modern wearable technologies face persistent limitations: limited battery life (1–7 days for continuous monitoring vs. target >30 days), poor skin adhesion (especially under sweating and motion), user discomfort from rigid materials, high costs (US$100–US$500 per device vs. target <US$50 for widespread adoption), and restricted functionality [2,34,66]. Critically, insufficient characterization of long-term stability (>3 months), reproducibility across manufacturing batches (typically ±15–30% variability), and mechanistic understanding of failure modes hinder clinical translation. These challenges underscore the need for more research, particularly interdisciplinary research, to bridge the gaps and advance wearable technology, including the exploration of new sustainable materials.

**Table 1:** State-of-the-art performance metrics for wearable technologies and target values.

Technology	Key Metrics	Current Performance	Target Values	Primary Limitations	Ref.
Graphene–metal hybrid sensors	Sensitivity; Conductivity; Stability	10.93 μA/mM/cm^2^; 1000–10,000 S/m; >100 cycles	>50 μA/mM/cm^2^; >10,000 S/m; >1000 cycles	Long-term drift; batch variability, ion/electron interface	
Flexible Li-ion batteries	Energy density; Cycle retention; Temp. range	100–200 Wh/kg; 70–80% after 500 cycles; 0–40 °C	>250 Wh/kg; >90% after 1000 cycles; −20 to 60 °C	Mechanical degradation, electrolyte stability	[42,43,44]
Solid-state batteries	Ionic conductivity; Safety	0.0001–0.0010 S/cm; High >0.01 S/cm; High	Low ionic conductivity at room temperature; interface resistance		
Textile supercapacitors	Energy density; Power density; Wash durability	<10 Wh/kg; 1–10 kW/kg; 70% after 10 washes	>50 Wh/kg; >5 kW/kg; >90% after 50 washes	Low energy density, washing-induced degradation, reproducibility	[45,46,47,48]
Thermoelectric generators	Power density; ZT value	44–156 μW/cm^2^; 0.8–1.2	>500 μW/cm^2^; >2.0	Small temperature gradients, thermal contact resistance, ambient dependence	[54,55]
Biofuel cells	Power density; Enzyme stability	1–5 mW/cm^2^; 50–70% after 1–2 weeks	>5 mW/cm^2^; >80% after 1 month	Enzyme degradation, metabolite availability, reproducibility	[56,57]
Dry electrodes (biosignals)	Impedance; SNR; Stability	10–100 kΩ; 5–10 dB lower than wet; 2–4 h	<10 kΩ; Equivalent to wet; >24 h	Motion artifacts, drift, contact quality	[59,60,61]
Conductive yarns (e-textiles)	Resistivity; Wash durability	0.01–100 Ω·cm; 50–80% after 10–20 washes	<0.01 Ω·cm; >90% after 50 washes	Washing degradation, mechanical failure, breathability reduction	[62,63,64,65]

### 2.6. Hydrogels as a Strategic Solution

Hydrogels—hydrophilic, crosslinked polymer networks with high water content (typically 70–95 wt% [67])—offer strategic advantages in addressing multiple wearable technology limitations. Their mechanical properties (Young’s modulus: 1–100 kPa) match those of human skin (4–20 kPa), facilitating conformal contact and reducing mechanical irritation. Ionic conductivity (0.01–0.1 S/m for salt-containing hydrogels) enables bioelectronic interfacing, while functionalization with carbon nanotubes or metal nanowires can enhance conductivity to 1–10 S/m [68,69,70,71]. Self-healing capabilities (recovery of 80–95% of mechanical properties within minutes to hours) and stimuli-responsiveness (e.g., thermoresponsive swelling/deswelling within 10–60 s) enable dynamic sensing and integrated drug delivery [72,73,74].

However, critical limitations must be acknowledged: (1) water loss: dehydration under ambient conditions (30–50% water loss within 24 h without encapsulation) compromises mechanical and electrical properties; (2) mechanical durability: fracture strain typically 100–500% vs. target >1000% for extreme body movements; tensile strength 10–100 kPa vs. target >500 kPa for robust wearables; (3) long-term stability: degradation of crosslinked networks over weeks to months, particularly under cyclic loading and varying pH/temperature; (4) reproducibility: hydrogel properties highly sensitive to synthesis conditions (gelation time, crosslinker concentration, temperature), resulting in batch-to-batch variability of ±10–20%; and (5) electron vs. ion conductivity trade-off: while hydrogels naturally support ion conductivity, achieving high electron conductivity (required for electronics interfacing) often compromises mechanical properties and biocompatibility.

Despite these limitations, hydrogels present a promising pathway for next-generation wearables. Section 3 and Section 4 detail hydrogel synthesis, properties, types, and recent advancements in hydrogel-integrated wearable systems, critically evaluating their performance against the target metrics outlined in Table 1 and assessing strategies to overcome current limitations in stability, reproducibility, and multifunctional integration. Collectively, these developments demonstrate the potential of hydrogels as a game changer for next-generation wearable devices, offering enhanced biocompatibility, mechanical adaptability, and functional integration for continuous, non-invasive health monitoring.

Noteworthy is that this review focuses on hydrogels, which differ from other gels. Hydrogels are three-dimensional (3D) crosslinked polymer networks with water as the continuous phase, characterized by high water content, which makes them highly biocompatible and suitable for direct skin-contact applications. In contrast, organogels contain organic solvents rather than water as the continuous phase, offering enhanced permeability for the delivery of materials (e.g., drugs) through lipophilic pathways and different mechanical properties suited to specific applications. Ionogels are gel systems in which ionic liquids serve as the continuous phase, enabling high ionic conductivity and electrochemical stability for bioelectronic and energy storage applications. These distinctions are critical when selecting materials for specific wearable applications: hydrogels excel at mimicking biological tissue properties and providing aqueous ion transport pathways, organogels enable enhanced small-molecule penetration for topical therapeutics, and ionogels facilitate high-speed electron transport for electronic applications.

## 3. Hydrogels—Synthesis, Properties and Types Explored for Wearable Technology

Hydrogels are typically soft, pliable, 3D polymer networks that absorb and retain fluids yet maintain their structural integrity. Crosslinking within the hydrogel network provides these 3D network structures and prevents dissolution when exposed to fluids [75]. Various components and techniques have been explored in the synthesis of hydrogels.

### 3.1. Hydrogel Synthesis

The synthesis process of a hydrogel significantly influences its structure, properties, and potential target applications. Understanding this relationship requires an in-depth knowledge of chosen synthesis methods. Hydrogels typically consist of polymers, crosslinkers, diluents, and/or initiators. They can be made from natural polymers (e.g., cellulose, chitin, hyaluronic acid, gelatin, agar, starch, alginate) or synthetic polymers (e.g., polyvinyl alcohol (PVA), polyacrylamide, polyethylene glycol (PEG)) [76,77]. Synthetic polymers are low in biodegradability and can release toxic substances into the environment, while natural polymers lack mechanical strength, require sterilization, and pose challenges for loading materials into the hydrogel cavity. The crosslinkers, which can be chemical (e.g., carboxylic acids, aldehydes) or physical (e.g., heat, ionic interactions), form bonds between polymer chains, creating a gel network. However, some chemical crosslinkers, like glutaraldehyde, have high toxicity [77]. Natural polymers are recently being explored to promote green processes [6]. Diluents, such as water and other solvents, control the rate of polymerization, and initiators, such as free radicals, UV light, or redox systems, trigger polymerization.

**Figure 2:**
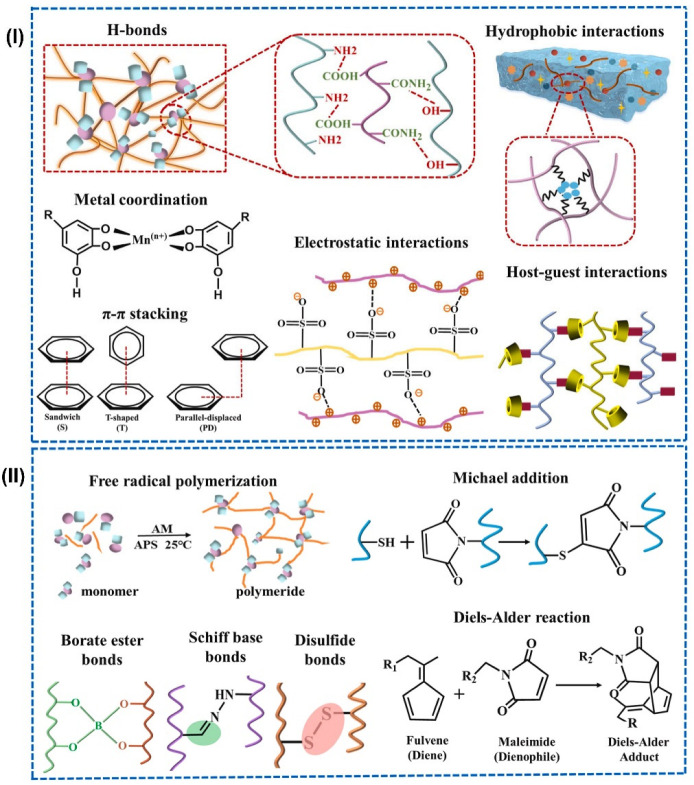
Hydrogel crosslinking methods, showing some (**I**) physical crosslinking and (**II**) chemical crosslinking strategies. Reproduced with permission from [80]. Copyright 2023, Elsevier.

Hydrogel synthesis steps involve selecting materials based on the desired hydrogel performance and properties, adding initiators to the polymer, and crosslinking. Crosslinking is crucial in hydrogel synthesis, as it affects key microstructural parameters, physicochemical properties, and end performance. The main crosslinking techniques are physical crosslinking, exploring techniques such as hydrogen bonding, hydrophobic interactions, electrostatic interactions, metal coordination, π-π stacking, crystallization, and intermolecular entanglement under certain conditions (Figure 2I); chemical crosslinking using techniques such as free-radical polymerization, condensation reactions, “click chemistry”, enzymatic reactions, irradiation and photopolymerization [78,79,80] (Figure 2II) or a combination of physical or chemical crosslinking mechanisms using multi-crosslinking techniques. Hydrogel crosslinking mechanisms, as well as fabrication techniques explored for wearables, are summarized below. The polymer–crosslinker mixture is then allowed to polymerize via bulk, solution, or suspension techniques, with solution processing preferred for better control of polymerization heat and industrial applicability. After polymerization, the sample is purified by washing with water or solvents to remove unreacted components or by-products, then dried using freeze-drying or vacuum drying. Lyophilization may be used before vacuum drying to remove water via sublimation, enhancing the porous structure. Finally, the crosslinked polymer network is hydrated to form the final hydrogel. The dried hydrogels can take various forms, including powders, particles, films, foams, beads, microspheres, patches, injectable liquids, and emulsions [6,81,82]. Details on hydrogel crosslinking strategies have been reviewed [80,83,84].

#### 3.1.1. Physically Crosslinked Hydrogels

Physical crosslinking is the formation of supramolecular hydrogels without the use of a crosslinking agent or external force. It utilizes non-covalent bonding interactions, such as ionic interactions, hydrogen bonds, crystallization, hydrophobic interactions, and other weaker physical forces, in the polymer network under certain conditions to form the hydrogel’s 3D network. Physical crosslinking can create either strong, permanent bonds (such as those found in glassy nodules or double and triple helices) or weak bonds that are reversible, breakable, reformable, or easily disrupted by stress or changes in physical conditions. Non-covalent bonding interactions are generally reversible, forming transient linkages. It is also linked to unstable, uncontrollable dissolution, difficulty in controlling hydrogel pore size, low mechanical resistance, and the formation of free chain ends or chain loops [85]. Physical crosslinking is influenced by various parameters, including polymer concentration, the molar ratio of each polymer, the type of solvent, the temperature of the solution, and the interaction between polymer functionalities. The amount and intensity of physically crosslinked interactions determine the mechanical properties (e.g., toughness and flexibility) of the hydrogel. The reversible non-covalent bonding of physically crosslinking hydrogels and their response to external stimuli enable their exploration for biodegradability, self-healing, and injectability at ambient temperatures.

##### Ionic Interactions

In ionic/electrostatic interactions, non-covalent bonds are formed between oppositely charged groups or metal–ligand interactions. The presence of counterions creates charged interactions that influence the hydrogel crosslinking. Polyanionic polymers and polycationic polymers are complexed to form hydrogels through complex coacervation [83], which exhibit rapid environmental response and self-healing properties. Coacervation is induced by modifications in pH, ionic strength, temperature, or solubility of the hydrogel media environment under controlled conditions.

##### Hydrogen Bonding

Hydrogen bonding is a widely used technique for crosslinking polymers containing polar functional groups, such as –OH and –COOH groups. When –COOH groups protonate in the presence of other polymers containing electron-deficient hydrogen atoms, hydrogen bonds can be formed [84]. In aqueous polymer systems with –COOH groups, hydrogen bonds form when pH is lowered. Acidic conditions reduce solubility, enabling hydrogen bonding. Natural polymers are largely explored for hydrogen bonding. Natural polymers (e.g., cellulose, chitosan, gelatin, and protein) contain abundant –OH and –COOH functional groups, which can interact with functional groups of other hydrogel components to produce hydrogen bonds.

##### Crystallization

The crystallization technique involves repeatedly freezing and thawing, during which amorphous and crystalline regions form within the hydrogel. The amorphous regions form when free water molecules occupy junctions or voids in the phase-separated material, while the crystalline regions form due to the aggregation of crystallites. The crystallites within a polymer chain can serve as physical crosslinking sites, facilitating the formation of hydrogels. A common polymer used for crosslinking via crystallization is polyvinyl alcohol (PVA). Crosslinking occurs at a temperature within the freezing range. The hydrogel properties, especially mechanical properties, are controlled by carefully tuning the degree of crosslinking between molecular chains or crystallinity of crystals during repeated freezing and thawing.

##### Hydrophobic Interactions

This is typically applied to water-soluble polymers incorporating hydrophobic groups or monomers, with thermal and ultrasonic processes aiding the formation of hydrophobic associations.

#### 3.1.2. Chemically Crosslinked Hydrogels

Chemical crosslinking refers to the formation of hydrogels through the use of a crosslinking agent to connect polymer chains within a polymer network, involving direct interactions between linear or branched polymers. It utilizes techniques such as free-radical polymerization, “click chemistry” (e.g., Diels–Alder, Schiff base, oxime, Michael-type addition, and boronate ester), and irradiation (gamma ray or electron beam) [78,79]. Initiators are also added to trigger the polymerization process. Chemical crosslinking enables the interaction of the functional groups (such as OH, COOH and NH_2_) with the selected crosslinking agent. Strong bonds are typically formed due to the permanent covalent bonds that are created between polymer chains, replacing hydrogen bonds with stable covalent bonds. Hence, chemically crosslinked hydrogels often exhibit improved mechanical stability, softness, and flexibility, as well as spatial and temporal control during gelation [6,86], compared to physically crosslinked hydrogels. However, most conventional hydrogels synthesized via chemical crosslinking contain toxic compounds as linking agents that can detach from the hydrogel during application or interact with loaded materials within the hydrogel. Chemical crosslinking is influenced by various parameters, including the type and concentration of monomer and crosslinker, solvent, reaction conditions employed, and exposure to heat, light, or an electron beam.

##### Free-Radical Polymerization

Free-radical polymerization is a common technique for synthesizing chemically crosslinked hydrogels. In this technique, vinyl monomers/polymers are polymerized and crosslinked with a crosslinking agent and initiators. The mechanical properties of the hydrogels are tuned by controlling monomer-to-crosslinker molar ratios. The most common monomers used include acrylic acid, acrylamide, and composite monomers containing natural macromolecules such as protein, gelatin, and chitosan [80]. Heat, light, radiation, and electron beams are applied to initiate free-radical polymerization.

##### Click Chemistry

“Click chemistry”, coined by Barry Sharpless, is commonly adopted to synthesize chemically crosslinked hydrogels with self-healing properties. Click reactions combine a C atom with a heteroatom X to form a C-X-C bridge to link polysaccharides, proteins or nucleic acids [84]. It includes Diels–Alder reaction, Borate ester bonds, Schiff base bonds, and Michael-type addition reaction. The Diels–Alder reaction yields hydrogels through the cycloaddition of dienes and/or dienophilic reagents, inducing complementary moieties for crosslinking. The Diels–Alder reaction produces no by-products or side reactions and requires neither a crosslinker nor an initiator. Due to its high atom efficiency, the lack of need for catalysts or initiators and its ability to proceed under mild conditions, it is considered an environmentally friendly technique for hydrogel synthesis [86,87]. Borate ester bonds are reversible covalent linkages formed via the condensation of boric acid or its derivatives with cis-diols. Hydrogels incorporating these bonds demonstrate rapid gelation, self-healing properties, and responsiveness to pH, temperature, and degradation [88,89]. Schiff base bonds arise from reactions between aldehyde- or ketone-containing compounds and amine-containing species, encompassing imine, hydrazone, and oxime linkages [90]. Hydrogels based on Schiff base chemistry typically exhibit pH sensitivity [90,91]. The oxime crosslinking produces hydrogels from reactions between the aminoxyl/hydroxylamine group and an aldehyde/ketone. Michael-type addition reactions enable the effective coupling of nucleophiles (Michael donors) with electron-poor olefins or alkynes (Michael acceptors) to create hydrogels [84]. It typically involves base-catalyzed addition of nucleophilic reagents (Michael donors, e.g., enolates, amines, thiols and phosphines) to active α, β-unsaturated carbonyl molecules (Michael acceptors, e.g., vinyl sulfones, acrylates, acrylonitrile and maleimides).

##### Irradiation

Irradiation is a versatile technique for hydrogel crosslinking that utilizes high-energy sources, e.g., gamma rays and ultraviolet (UV) light, to initiate polymer network formation. This process generates free radicals within polymer chains or monomer solutions, forming covalent bonds and a stable three-dimensional hydrogel matrix. Irradiation can proceed without the use of chemical initiators or crosslinking agents [78], thereby reducing the risk of cytotoxicity—a crucial consideration for applications involving direct skin contact. The extent of crosslinking can be precisely modulated by adjusting parameters such as radiation dose and exposure time, enabling tuning of key hydrogel properties (e.g., mechanical strength, swelling, and electrical conductivity). These tunable characteristics are particularly beneficial for wearable technologies, where hydrogels are required to exhibit flexibility, durability, and biocompatibility. When combined with conductive fillers, irradiation-crosslinked hydrogels can also support electronic functionality, making them ideal for use in biosensors, strain gauges, and epidermal electronics. Moreover, irradiation could enable the direct integration of hydrogel functionalities onto or within fabric substrates, creating hybrid materials that respond dynamically to environmental or physiological stimuli. For example, UV or gamma irradiation can be employed to graft hydrogel networks onto textile fibers, imparting properties such as moisture responsiveness, thermal regulation, flame resistance, or controlled drug release [92]. This approach supports the development of smart garments for medical, athletic, or protective applications. Furthermore, the non-thermal nature of irradiation preserves the structural integrity of delicate textile materials while enabling spatial control over crosslinking. This enables the fabrication of textiles with localized sensing or actuation capabilities, such as pH-sensitive drug delivery zones or temperature-responsive ventilation panels. The resulting materials offer multifunctionality and adaptability, positioning irradiation-crosslinked hydrogels as a cornerstone in the advancement of next-generation wearable and textile-based systems.

#### 3.1.3. Multi-Crosslinked Hydrogels

With the growing demand for advanced hydrogel applications, single-mode physical or chemical crosslinking often fails to meet performance requirements. Consequently, multi-crosslinking strategies have emerged, integrating one or more physical and/or chemical crosslinking mechanisms to enhance the functional properties of hydrogels [93,94,95,96,97,98]. Physically crosslinked hydrogels offer excellent biocompatibility, while chemically crosslinked ones provide superior mechanical strength. Their combination yields hybrid crosslinked hydrogels with improved toughness, elasticity and self-healing properties.

#### 3.1.4. Fabrication Techniques Employed for Optimizing Hydrogel Performance

Fabrication techniques such as three-dimensional (3D)/four-dimensional (4D) printing, electrospinning, and microfabrication have been employed to develop hydrogels with tailored properties that meet the specific needs.

3D or 4D printing: Three-dimensional (3D) printing method involves extruding a hydrogel precursor through a nozzle to fabricate structures layer by layer. It enables rapid prototyping, fabrication of complex geometries, and compatibility with a wide range of hydrogel materials. The grid-like microstructure of 3D-printed hydrogel sensors enhances their sensitivity. Such printed hydrogels have been widely investigated for wearable sensing applications [99,100,101,102,103,104], where hydrogels are embedded with conductive materials and printed into flexible sensors for health monitoring. For example, Ren et al. [99] prepared a sodium alginate/sodium polyacrylate/layered rare-earth hydroxide (LRH) nanocomposite hydrogel through ionic and coordination crosslinking, which was subsequently used as a 3D printing ink to produce intricate, highly precise structures. The hydrogel exhibits excellent 3D printing performance at room temperature and tunable fluorescence emission, with potential applications in sensing. Hao et al. [101] prepared a skin-inspired hydrogel using PVA, sodium alginate, glycerol and silver nanowires and fabricated it into complex structures (e.g., dumbbells, multilayer rectangular squares and grid structures), exploring 3D printing as a method. The 3D-printed hydrogel exhibits high tensile strength, excellent recovery and fatigue resistance under yield strain. It can also be used for detecting human motion signals. Wu et al. [102] developed a multifunctional hydrogel suitable for digital light processing (DLP)-based 3D printing in wearable sensing applications. By incorporating both physical and chemical crosslinking networks using polymers such as poly(acrylamide), poly(acrylic acid), poly(ethylene glycol) diacrylate, silk fibroin, and glycerol, the hydrogel achieved superior mechanical strength, remarkable adhesion (maintained even after 10 days of storage), and excellent water retention. The resulting sensor demonstrated a gauge factor of 1.29 under tensile strains from 1% to 100%, along with a stable relative resistance change across 80 cycles.

Four-dimensional (4D) printing refers to creating 3D-printed structures—often regarded as smart, adaptable materials—that can respond predictably to external stimuli such as heat, moisture, pH, light, water, and magnetic or electric fields [100]. Compared with conventional 3D printing, it offers notable benefits, including greater design flexibility, accelerated production, reduced costs and material waste, and a streamlined one-step manufacturing process. Innovative materials employed for 4D printing include polymers with excellent mechanical and functional properties, such as polylactic acid, polycaprolactone and polyethylene glycol, ceramics and alloys. Some printing techniques explored include inkjet printing and stereolithography. Inkjet printing utilizes droplets of hydrogel precursor that are deposited onto a substrate. It is suitable for creating fine features and patterns. Stereolithography (SLA) involves the use of light to polymerize a hydrogel precursor layer in a layer-by-layer process. SLA offers high resolution and precision, making it ideal for creating detailed microstructures. Discussions on 3D/4D printing of soft materials, including hydrogels and the printing techniques explored, are available [105,106].

Electrospinning: This involves applying a high voltage to a polymer solution to create a jet that solidifies into fine fibers (diameters ranging from nanometers to micrometers) as it travels toward a grounded collector. The choice of polymer solutions and additives for electrospinning is important. The choice of polymer and solvent affects the fiber morphology and mechanical properties. Common polymers include polyvinyl alcohol (PVA) and polycaprolactone (PCL). Incorporating additives such as nanoparticles and bioactive agents into the polymer solution can enhance the functionality of the electrospun fibers. Electrospinning has been explored to develop hydrogel-based smart wearable textiles. Electrospun hydrogel fibers have been woven into fabrics to create moisture-wicking and breathable textiles. Due to their significant surface area and porosity, electrospun fibers are attractive for wearable devices that deliver functional agents.

Microfabrication: Among microfabrication techniques explored for wearables are photolithography and soft lithography. Photolithography involves coating a substrate with a light-sensitive photoresist, exposing it to light through a mask, and developing the exposed areas to create micro-scale patterns. It has been used to create microfluidic devices and sensors with precise control over the hydrogel microstructure. Soft lithography, including microcontact and replica moulding, involves creating a mould from a master pattern and using it to shape the hydrogel precursor. Soft lithography is suitable for creating flexible and stretchable electronics.

### 3.2. Hydrogel Properties Essential for Smart Wearable Devices and Textiles

The integration of wearable devices and textiles with the human body necessitates materials that can simultaneously meet the demands for functional performance and physiological compatibility. As wearable technologies evolve toward continuous, real-time health monitoring and therapeutic applications, the interface between the wearable and the skin becomes increasingly critical. Materials used in this interface should be soft, conformable, and biocompatible while also supporting advanced functionalities such as sensing, actuation, and data transmission. Hydrogels stand out among candidate materials for their unique blend of mechanical flexibility, chemical versatility, and biocompatibility. This section outlines the key hydrogel characteristics relevant to wearable technologies, distinguishing between inherent properties—those naturally arising from the hydrogel’s structure—and engineered or enhanced properties—those introduced through material modification or composite design. Inherent properties include flexibility and stretchability, wet adhesion, biocompatibility, skin conformability, permeability, and, in many cases, optical transparency. Engineered properties include electrical conductivity, self-healing, tunable mechanical properties, and stimuli-responsiveness, which expand their functional scope, allowing them to serve as active components in smart wearables.

Flexibility and stretchability: This is the ability of a material to bend, twist, or elongate without breaking. Hydrogels exhibit high deformability, allowing them to stretch, bend and twist in response to body movements without mechanical failure. The flexibility and stretchability of hydrogels are attributable to their loosely crosslinked polymer networks, which are swollen with water, allowing polymer chains to slide and rearrange under stress. These characteristics enable the hydrogel to conform to complex body contours and accommodate natural movements, reducing mechanical mismatch. Flexibility and stretchability are crucial for maintaining accurate signal acquisition and user comfort. Noteworthy is that excessive swelling could compromise mechanical strength. Therefore, optimizing hydrogel crosslinking density is critical for achieving optimal flexibility and stretchability in wearable applications. Materials explored for flexible and stretchable hydrogels include glycerol, polyvinyl alcohol (PVA), polyethylene glycol (PEG), polyacrylamide (PAAm), poly(N-isopropylacrylamide) (PNIPAm) and ionic salts [101,102,107].

Wet adhesion: This refers to a material’s ability to adhere to moist or wet surfaces, such as human skin. Unlike traditional adhesives, hydrogels can adhere to moist or sweaty skin through hydrogen bonding, van der Waals interactions, and sometimes, covalent bonding with skin proteins. This wet adhesion is essential for maintaining stable contact during physical activity or prolonged wear, particularly in epidermal sensors and patches. It is essential that the hydrogel’s wet adhesion balances strength and removability to avoid skin damage or discomfort. Materials explored for wet adhesive hydrogels include adhesive moieties such as catechol groups, tannic acid, dopamine, gelatin, chitosan and hyaluronic acid [107,108,109,110].

Biocompatibility and skin conformability: Biocompatibility refers to the “ability of a material to perform with an appropriate host response in a specific application” [111] without eliciting adverse effects such as toxicity, irritation, or immune response [6]. Hydrogels naturally fulfill this requirement due to their high water content, soft and rubbery tissue-like consistency and hydrophilic surface, which collectively reduce interfacial energy and mechanical mismatch with skin. These properties minimize irritation and inflammation, making hydrogels ideal for continuous skin contact and even implantable devices. In parallel, their softness and conformability—attributable to low crosslinking density and high hydration—allow hydrogels to mould intimately to the skin’s microtopography, ensuring stable contact and user comfort. For wearable applications, it is essential that hydrogels maintain structural integrity while remaining non-toxic, non-immunogenic and free from leachable harmful substances. To further enhance these properties, bio-based materials, such as silk fibroin, gelatin, cellulose, chitosan, and alginate, have been explored for biocompatibility, offering additional benefits in terms of sustainability and biodegradability [3,102]. Some non-toxic, FDA-approved synthetic polymers include polyethylene glycol and polyvinyl alcohol. Softness and skin conformability have been improved using low-crosslinked polymers such as PAAm, poly(ethylene glycol) diacrylate (PEGDA) and plasticizers like glycerol and sorbitol.

Permeability: This refers to a material’s ability to allow the passage of gases, water vapour, or small molecules through its structure. In hydrogels, this property arises from their porous polymer network, hydrophilic nature, and, in some cases, the presence of labile bonds that facilitate molecular diffusion. At high swelling levels, certain labile bonds may begin to dissociate, leading to partial degradation or dissolution of the hydrogel matrix. The degree of resistance to dissolution is primarily governed by the density and stability of crosslinks within the polymer network. Permeability is a critical parameter for wearable applications, particularly those involving skin contact, biochemical sensing, or controlled release. For instance, breathable hydrogels support skin respiration and enable the diffusion of sweat and analytes, which is essential for applications such as sweat-based diagnostics. In contrast, impermeable materials can trap moisture and heat, leading to discomfort, irritation, and reduced sensor performance due to sweat accumulation and skin occlusion. The interconnected microporous structure of hydrogels not only facilitates the efficient transport of heat and moisture but also contributes to their mechanical softness and deformability. This dual functionality makes hydrogels ideal for developing breathable, skin-conforming wearable devices and smart textiles. However, hydrogel permeability must be carefully balanced—excessive permeability can lead to dehydration and loss of structural integrity, while insufficient permeability may hinder comfort and sensor accuracy. Therefore, tailoring pore size, crosslinking density, and polymer composition is essential to optimize hydrogel performance for specific wearable applications. Materials explored for permeable hydrogels include alginate, gelatin, silicone, metal-coated nanofiber mesh coupled with PEDOT:PSS [112], and PEG.

Optical transparency: This refers to the optical clarity of a material in the visible spectrum. This is achieved by avoiding phase separation and using optically clear polymers. Optically transparent hydrogels are advantageous for optical sensing and imaging applications. Transparency also allows for visual inspection of the skin beneath the wearable device, especially essential for clinical monitoring. Transparent hydrogels can be designed by optimizing hydrogel composition to enhance crystallinity and uniformity. Transparency should not compromise mechanical or functional performance. PEG, PVA and PAAm are among the optically clear polymers explored [113,114].

Electrical conductivity: This refers to the ability to conduct electrical current. Hydrogel conductivity is achieved by incorporating conductive fillers, metallic nanowires (e.g., silver nanowires), Mxenes, or using intrinsically conductive polymers, including PEDOT:PSS, polyaniline (PANI), and polypyrrole (PPy) [115,116,117,118,119]. Conductive hydrogels enable the development of bioelectrodes and sensors that can transmit physiological signals with high fidelity. It is essential to maintain conductivity under deformation and over time; uniform dispersion of conductive elements is critical.

Self-healing: This refers to the ability of a material to autonomously repair mechanical damage. Self-healing hydrogels can autonomously repair mechanical damage through reversible physical interactions or chemical bonds such as hydrogen bonding, ionic interactions or dynamic covalent bonds (e.g., Schiff base and disulfide bonds). This property enhances device durability and longevity, especially in dynamic environments where mechanical stress is frequent. The healing speed and efficiency of hydrogels depend on environmental factors, such as temperature and humidity. Self-healing hydrogels have been developed using materials such as aldehyde-modified hyaluronic acid (AHA), aminated diselenide crosslinker 2,2′-diselanediyldiethanamine dihydrochloride (Sel) with diselenide bonds, aliphatic polycarbonate, sodium alginate/polyacrylamide, carboxymethyl chitosan/acryloyl-6-aminocaproic acid, carboxymethyl chitosan/poly(acrylic acid), and PEG functionalized with boronic acid [120,121].

This self-repairing capacity greatly enhances device durability and longevity, particularly in dynamic environments where wearable devices and textiles are subjected to frequent mechanical stress, such as bending, stretching, or impact. The healing speed and efficiency of these hydrogels depend on factors like temperature and humidity, with optimized matrices showing rapid recovery of both structure and electrical function after damage. For real-time monitoring applications, the restoration of conductivity and sensor integrity is essential [122,123]: when cracks or tears occur in the hydrogel electrode or sensor layer, dynamic interactions or reversible bonds reconnect the polymer network, restoring reliable measurement of biosignals or analyte concentrations without the need for manual intervention. Beyond mechanical resilience, self-healing hydrogel wearables provide protective benefits. Water-rich, elastic interfaces shield the skin while preserving breathability, minimizing irritation during continuous use. When combined with antimicrobial or UV-protective additives, self-healing hydrogels further safeguard users against environmental hazards and infection. Incorporating conductive fillers like carbon nanotubes or PEDOT:PSS allows both physical and electrical self-repair, vital for uninterrupted performance in health diagnostics, movement tracking, and smart textile systems. Examples of hydrogel-based wearables integrating self-healing properties are discussed in Section 4 [124,125,126].

Tunable mechanical properties: This refers to the ability of a material’s mechanical properties to be tuned. Hydrogels can be designed to have adjustable stiffness, toughness and elasticity by modifying their polymer composition, crosslinking density and incorporating reinforcing agents. This tunability allows customization for specific application requirements. Trade-offs between hydrogel mechanical strength and flexibility should be optimized. Materials explored include crosslinking agents like N, N’-methylenebisacrylamide (BIS), genipin, UV or ionic crosslinking and reinforcing materials like nanoclay, PAM, carboxymethyl chitosan [127], cellulose nanocrystals, and microfibrillated cellulose [128,129].

Stimuli response: This is a material’s ability to change properties in response to external stimuli. Hydrogels can respond to temperature, pH, light, electric fields and other external stimuli. Their stimulus response is attributable to the presence or incorporation of functional groups or responsive moieties that undergo conformational or chemical changes in their microstructure. These responsive behaviours enable dynamic sensing, controlled release of functional agents and adaptive interfaces in wearable systems. For wearable devices and textiles, hydrogel responsiveness should be reversible, repeatable and compatible with physiological conditions. Thermoresponsive polymers (e.g., PNIPAm, hydroxypropyl cellulose), pH-responsive polymers (e.g., polyacrylic acid and chitosan), light-responsive polymers (e.g., azobenzene), and electric field-responsive materials (e.g., polyelectrolytes) have been explored for stimuli-responsive hydrogels [130,131].

### 3.3. Hydrogel Types Applicable for Wearables

#### 3.3.1. Conductive Hydrogels

Conductive hydrogels, unlike conventional hydrogels, possess intrinsic electrical conductivity. Their electrical properties are primarily governed by the nature of their polymeric constituents and the type of conductive materials incorporated. Based on their conductive components, conductive hydrogels have been classified into three categories: (1) ionic conductive hydrogels, which incorporate ionic liquids and electrolytes; (2) nanocomposite conductive hydrogels, which include ionized nanotubes or inorganic conductive fillers; and (3) polymeric conductive hydrogels, which are based on intrinsically conductive polymers [132].

These hydrogels exhibit high electrical conductivity, as well as tunable physicochemical properties and responsiveness to external stimuli [133,134,135,136], making them highly promising for smart wearable devices and textiles. Potential uses include smart sensors, energy storage and conversion, health monitoring and human–computer interfaces [137].

##### Ionic Versus Electronic Conductivity Mechanisms

The electrical conductivity of conductive hydrogels arises from two primary mechanisms—ionic conduction and electronic conduction [132,138]. These mechanisms differ fundamentally in their charge-carrier types, transport pathways, and material compositions, making them suitable for different wearable applications. Understanding these mechanisms is essential for selecting appropriate hydrogels for specific wearable applications.

Ionic conduction involves the migration of cations and anions through the three-dimensional hydrogel network via electrostatic interactions and hydration effects. In these hydrogels, ions move through interconnected aqueous pathways created by polymer chains and their interstitial spaces, mimicking biological ion transport mechanisms. This approach is particularly valuable for bioelectronic applications such as bioelectrodes and biosensors, where intimate contact with biological tissues is required. Ionic hydrogels demonstrate excellent ionic conductivity (up to 51.48 S/cm), water retention, mechanical elasticity, optical transparency, and anti-freezing capabilities [139,140].

Electronic conduction is achieved by embedding conductive electronic components within the polymer matrix to form continuous electron transport pathways. This mechanism depends on the transport of electrons or holes through the material via π-electron delocalization in conjugated polymer backbones. Electrons move efficiently through hopping or tunnelling mechanisms, enhanced by the formation of percolation pathways—continuous networks of interconnected conductive components that facilitate electron mobility. Conjugated polymers such as polyaniline (PANI), polypyrrole (PPy), and poly(3,4-ethylenedioxythiophene) (PEDOT) achieve high conductivity (10–1000 S/cm for PEDOT:PSS) through this mechanism. Electronic conduction enables faster signal transmission and higher overall conductivity, making it advantageous for applications requiring rapid electronic response times [132,141].

Thus, ionic hydrogels preserve inherent soft material properties and excel at biocompatibility, while electronic conduction may enhance mechanical robustness through integration of rigid conductive fillers. Advanced hydrogels employ dual-conductive mechanisms, combining both ionic and electronic pathways to optimize performance for hybrid applications—for example, achieving both lithium-ion conductivity (0.000276 S/cm) and electronic conductivity (68.9 S/cm) [142]. Carbon-based materials with sp^2^-hybridized carbon atoms (graphene, carbon nanotubes) enable particularly efficient electron transport; single-walled carbon nanotubes can achieve conductivity of 100 to 1,000,000 S/cm, while graphite with perpendicular atomic arrangements exhibits lower conductivity [132].

##### Classification of Conductive Hydrogels

Based on their conductive mechanisms and materials, conductive hydrogels are classified as ionic conductive hydrogels, electroconductive hydrogels, and metal-based conductive hydrogels [132,143,144].

Ionic conductive hydrogels feature repeating cationic and anionic groups within a 3D network with interconnected pores enabling ion transport. The conductivity mechanism arises from molecular-scale ion channels formed by specific ion coordination—for example, coordination between alginate G-block units and lithium ions facilitates directional ion migration under electric fields. Hydration and swelling behaviours further modulate conductivity by enlarging free volume for ion movement. These hydrogels demonstrate excellent ionic conductivity, water retention, mechanical elasticity, optical transparency and anti-freezing capabilities [133,134].

Electroconductive hydrogels integrate electroconductive materials (polypyrrole, polyaniline, carbon nanotubes) with polymeric matrices such as polyvinyl alcohol [145,146]. Their conductivity derives from delocalized π-electron systems within conjugated polymer backbones, enabling efficient electron transport.

Metal-based conductive hydrogels utilize the superior electrical and mechanical properties of metals (gold, silver, platinum, palladium) to enhance overall performance [132]. These metal nanoparticles are incorporated through crosslinkers, precursors, or direct integration, forming conductive networks that enhance electron transport with conductivity values ranging from 0.3 to 0.8 S/cm (gold) to 0.5–0.7 S/cm (silver).

##### Conductive Materials and Hydrogel Network Formation

Various conductive materials have been explored for developing hydrogels, including conductive polymers, carbon-based materials, metal nanoparticles, and hybrid materials [147].

Conductive polymers (polyaniline, polypyrrole, PEDOT, polyacetylene, polythiophene) conduct electricity through delocalized π-electron systems within their conjugated backbones. Advanced techniques such as doping or compositing can significantly enhance their conductivity.

Carbon-based materials (carbon nanotubes, graphene, carbon dots, fullerenes) achieve high conductivity through sp^2^ hybridization of carbon atoms, enabling efficient electron mobility. In parallel atomic arrangements, electrons move freely between layers, achieving conductivity of 100 to 1,000,000 S/cm, while perpendicular arrangements (as in graphite) exhibit lower conductivity. These materials form essential percolation networks within the hydrogel matrix, creating continuous pathways for electron transport [132].

Hybrid materials combine natural and synthetic polymers (polysaccharides, cellulose, alginate, polyvinyl alcohol) with conductive agents (polypyrrole, PEDOT), tailoring mechanical and physicochemical properties while maintaining high conductivity and biocompatibility.

##### Self-Healing Conductive Hydrogels

Despite their advantages, conductive hydrogels face challenges, including limited operating temperature ranges, suboptimal mechanical strength, and difficulty restoring conductivity and mechanical integrity after damage [147,148]. Self-healing conductive hydrogels address these limitations by restoring both electrical and mechanical functionality.

Self-healing mechanisms (briefly introduced in Section 3.2) operate through non-covalent interactions, including hydrogen bonding, van der Waals forces, and π-π stacking, enabling rapid recovery at room temperature without external stimuli [149]. Mechanically and electrically self-healing hydrogels incorporating multi-walled carbon nanotubes (MWCNTs) can recover mechanical properties and electromagnetic interference (EMI) shielding effectiveness within 7 days, with self-healing efficiency reaching 77.2% [150]. The synergistic effect of recombination of hydrophobic interactions and the readsorption of polymer chains onto MWCNT and cellulose nanofiber surfaces enables simultaneous recovery of mechanical damage and electrical conductivity. Advanced formulations achieve 80–95% recovery of mechanical properties within minutes to hours through cooperative dynamic interactions [149].

These self-healing conductive hydrogels restore interior structure, mechanical properties, electrical conductivity, and EMI shielding performance after mechanical damage, enhancing the durability, cost-effectiveness, and environmental sustainability of wearable devices. The ability to simultaneously restore electrical and mechanical functionality is particularly valuable for long-term wearable applications where device integrity is critical to continuous performance. Composite hydrogels with enhanced stretchability, compressibility, adhesion, anti-freezing properties, water retention, and antibacterial activity represent the current frontier in conductive hydrogel development.

#### 3.3.2. Natural Polymer-Based Hydrogels

Natural polymer-based hydrogels play a transformative role in advancing wearable technology, particularly due to their biocompatibility, flexibility, and responsiveness to stimuli. Natural polymer-based hydrogels are increasingly investigated in wearable devices for flexible sensors, drug delivery systems, wound monitoring, sweat and motion detection and bioelectronic interfaces. There is also a desire to develop environmentally sustainable wearables, which has led to the exploration of natural polymers such as those based on cellulose [151,152,153] and chitin [154] and their derivatives [155,156], due to their natural abundance and non-toxicity. The strategic importance of natural polymers extends beyond simple availability; they offer clear biocompatibility advantages, biodegradability, and low environmental impact, addressing the growing demand for sustainable wearable devices and textiles.

##### Cellulose-Based Hydrogels

Cellulose is the most abundant natural polymer on earth, providing a low-cost and sustainable resource that is renewable, degradable and biocompatible. Cellulose consists of linear chains of repeating β-D-glucopyranose units covalently linked through β-1,4 glycosidic bonds, with a large number of hydrogen bonds existing intra- and inter-molecularly to produce different configurations of cellulose structure [4,157]. The structural and chemical features of cellulose, including transparency, low thermal expansion, anisotropy, high elasticity, and flexible surface chemistry, have enabled the functionalization and engineering of cellulose-based hydrogels for wearable technology [151,152,153,158], as discussed in Section 4. During cellulose dissolution, intramolecular hydrogen bonds are broken, and the supramolecular structure is disrupted, enhancing the activity of hydroxyl groups and enabling easier combination with other natural or synthetic polymers by reconstructing hydrogen bonds. Cellulose-based hydrogels endow specific performance, such as biodegradability, renewability, flexibility, and high mechanical strength. Cellulose-based hydrogels have also been explored for their adaptability to mechanical and anti-freezing properties, even at sub-zero temperatures [159].

##### Polysaccharide-Based Hydrogels

Other natural polymers commonly explored for wearables include polysaccharides [160,161,162,163,164,165,166] such as chitosan, alginate and starch, as well as less-explored glycosaminoglycans [167] such as hyaluronic acid and chondroitin sulfate.

Chitosan has been explored for its antimicrobial properties and biodegradability in biosensing applications. As the only cationic polysaccharide found among natural polysaccharides, chitosan is well known for its antimicrobial and antifungal properties, enabling interactions with negatively charged surfaces of cells and microbes. Chitosan exhibits degradability in vivo by human proteases such as lysozyme due to its multiple amino groups. Recent studies have highlighted the antitumor activity, mucoadhesive, and hemostatic properties of chitosan. However, the applications of chitosan-based hydrogels in wearable technology have been limited by insufficient mechanical strength and inadequate degradation rates, necessitating the preparation of composites by mixing chitosan with other functional substances to improve performance.

Alginate has been studied in flexible sensors and delivery systems. Alginate is a naturally available biopolymer and a linear anionic polysaccharide consisting of repeated residues of α-L-glucuronate (G) and β-D mannuronate (M) [168]. It provides biocompatibility, biodegradability, non-antigenicity, chelating ability, and good stability for extended periods. The mechanical properties of alginate hydrogels formed through ionic crosslinking with divalent cations (e.g., Ca^2+^, Ba^2+^, Zn^2+^) are directly related to the concentration of the crosslinking cation and the molecular weight and composition of G-blocks. Although alginate is biocompatible, biodegradable, and non-toxic, it has several disadvantages, such as low bioadhesivity and biological inertness, which limit its applications in smart wearables. The partial oxidation of alginate chains is an attractive method to make alginate degradable under physiological conditions by altering the chain conformation to enable backbone degradation.

Starch is abundant and biodegradable and is being explored for eco-friendly wearable substrates. Starch is a natural polysaccharide composed of glucose repeating units linked through α-D-(1-4) and α-D-(1-6) glycosidic linkages, with a wide range of applications due to its low cost, renewability, biodegradability, and biocompatibility [159]. Rich in hydroxyl groups, starch has excellent hydrophilic properties and is an ideal candidate for developing hydrogels with higher swelling capacity and enhanced biodegradability. Modified starch, such as ozonated cassava starch, can be processed into hydrogels with improved properties, including better pasting properties and printability.

Hyaluronic acid (HA), with its excellent moisture retention and biocompatibility, has been explored for skin-contact applications. As a main component of the extracellular matrix found prominently throughout the body, hyaluronic acid comprises N-acetyl-glucosamine and D-glucuronic acid residues [169]. It is well known for its bioactivity and plays significant roles in lubrication, water absorption and retention, and structural functions, while interacting with various cell receptors to coordinate cell communication and behaviour. Because of its nontoxicity, non-allergenicity, biocompatibility, and biodegradability, hyaluronic acid has been widely used as a biomedical material. Hyaluronic acid degrades rapidly in the body and is often used in combination with other materials to extend retention time in vivo. The chemical modification of hyaluronic acid focuses on distinct functional groups, including glucuronic carboxylic acids, primary and secondary hydroxyl groups, and N-acetyl groups.

##### Limitations of Natural Polymer-Based Hydrogels

Natural polymer-based hydrogels show limitations in mechanical strength and degradation control. To overcome these limitations, they are often subjected to chemical modifications, crosslinking, or blending with other natural polymers or synthetic polymers to form composite hydrogels. For example, alginate dialdehyde–gelatin hydrogels show higher degradability compared to alginate alone and exhibit good cell adhesion, proliferation, and migration properties. Although natural polymers can increase the degradation rate of hydrogels in general, the reaction employed can control the degradation rate of the material. The presence of ester groups allows ease of degradation, whereas biomolecules with aldehyde and carboxylic acid functional groups show decreased degradation [170]. Degradation depends not solely on certain functional groups but on the interactions between biomolecules—strong conjugation leads to slow or limited degradation, while weak conjugation or free polymeric chains can disrupt hydrogen bonding, rendering chains more mobile and decreasing water-holding capacity.

#### 3.3.3. Composite Hydrogels

Composite hydrogels represent a sophisticated design strategy to overcome the inherent limitations of single-component hydrogels by combining multiple materials to achieve synergistic improvements in electrical conductivity, mechanical properties, flexibility, adhesion, self-healing, and biocompatibility. The fundamental approach involves incorporating nanoparticles, conducting polymers, carbon-based materials, metal nanoparticles, and reinforcing fibers into hydrogel matrices, creating multifunctional materials particularly suitable for biosensors, bioelectronics, flexible sensors, and wearable device applications. This approach explores the synergistic effects of different components.

##### Component Integration and Design Strategy

Composite hydrogels strategically combine diverse primary components (conducting polymers, carbon-based materials and metal nanoparticles) to exploit the strengths of each material [171]. Conducting polymers such as polyaniline and poly(3,4-ethylenedioxythiophene) (PEDOT) enhance electronic conductivity through delocalized π-electron systems while maintaining flexibility, softness, and biocompatibility essential for user comfort. Carbon-based materials, including graphene oxide and carbon nanotubes, improve both electrical conductivity and mechanical strength through percolation network formation. Metal nanoparticles (gold, silver, platinum, palladium) further enhance electrical conductivity and impart antibacterial properties. Reinforcing nanofibers such as cellulose nanofibers (CNF) create mechanical reinforcement networks through hydrogen bonding and physical entanglement with the polymer matrix.

The key innovation involves in situ polymerization of conducting monomers within the hydrogel matrix, ensuring uniform distribution of conductive materials while enabling fine-tuning of hydrogel properties through adjustable polymerization conditions. This approach produces meta-composites with robust, durable properties that integrate the strengths of each component—electronic conductivity from conjugated polymers, mechanical stability from nanofibers and reinforcing materials, and biological functionality from natural polymers and metal nanoparticles.

##### Natural and Natural Polymer (Biopolymer-Based) Composites

Natural polymers are combined to enhance properties and achieve multifunctionality while maintaining the environmental sustainability of the hydrogels. Recent advances have demonstrated multiresponsive natural polymer composite hydrogels that respond to multiple environmental stimuli, including temperature, humidity, strain, and light, offering high transparency and biocompatibility for real-time biosensing and wearable e-skin applications. For example, alginate–gelatin hydrogels achieve multiresponsiveness through ionic crosslinking with multivalent cations, enabling responsive behaviour across diverse environmental parameters [172]. The combination of sodium alginate and gelatin provides an excellent hydrogel substrate due to its unique biological properties, including biocompatibility, biodegradability, and non-toxicity. The obtained alginate–gelatin crosslinked hydrogel (ADA-GEL, formed through covalent crosslinking of alginate dialdehyde and gelatin via Schiff base formation) can be used to produce hydrogels with good mechanical strength and biocompatibility for regenerative medicine applications [173]. Cellulose derivatives and chitosan have also been explored for the development of thermoresponsive hydrogels with excellent hydrolytic stability [6]. Chitosan–alginate bilayer hydrogels exemplify synergistic electrostatic interactions. Chitosan, derived from chitin through deacetylation, becomes positively charged in acidic environments (pH < 6.5) due to its protonated amino groups. Alginate, a polysaccharide from brown seaweed featuring a negatively charged backbone (carboxyl functional groups), remains water-soluble and forms gels through ionic crosslinking with divalent cations such as Ca^2+^. The electrostatic attraction between positively charged chitosan and negatively charged alginate significantly enhances hydrogel adhesion to biological tissues and skin, creating bilayer structures with improved overall performance. This combination allows independent tuning of individual layer properties while maintaining integrated functionality.

##### Natural and Synthetic Polymer Composites

Natural polymers are also combined with synthetic polymers—including cellulose derivatives, gelatin, chitosan, hyaluronic acid, and alginate—selected for their inherent biocompatibility and bioactivity. Synthetic polymers—such as polyvinyl alcohol (PVA) and polyethylene glycol (PEG)—provide tunable mechanical properties and controllable degradation rates. The combination of these components creates hydrogels with precisely engineered functionality. For example, alginate–PVA composites leverage alginate’s biocompatibility and stimuli-responsiveness combined with PVA’s mechanical strength and thermal stability, creating flexible yet robust materials suitable for long-term wearable applications. Bilayer hydrogels with Janus characteristics have also been developed, combining gelatin and modified polyvinyl alcohol (PVA) through dynamic interfacial bonding via Schiff-base bonds and hydrogen bonding at ambient conditions [172]. These bilayer hydrogels exhibited high stretchability, flexibility, and toughness, with the PVA-rich layer contributing to mechanical strength and water retention while the gelatin-based layer provided softness and biocompatibility. Mechanical properties can be fine-tuned by adjusting polymer concentration, crosslinking time, and functional group density. Anisotropic swelling—where one layer swells more than the other upon water absorption—enabled directional actuation and programmable, stimuli-responsive behaviour with controlled shape transformation.

Recent advances demonstrate that composite hydrogel design can achieve exceptional mechanical properties through multi-network architectures [171,174]. A notable example is the triple-network interlocked structure developed by combining PVA/polyacrylamide (PAM) dual-network hydrogels with cellulose nanofibers (CNF) [171]. The CNFs form numerous hydrogen bonds and entanglements with the polymer matrix, with their hydroxyl groups interacting with the PVA network to create a third conductive network. This triple-interlocking integration creates multiple energy-dissipation pathways, resulting in composite hydrogels with mechanical strengths of 1.41 MPa and toughness of 6.73 MJ/m^3^ after a single freeze–thaw cycle. Coordination between Zn^2+^ ions and carboxyl groups on CNF further reinforces network interlocking, imparting exceptional electrical conductivity, antibacterial properties, high transparency (89.8%), sensitivity, and biocompatibility—making such materials promising for biosensing and flexible electronics. Double-network hydrogels consisting of a stiff first network interpenetrated with a flexible second network overcome the mechanical limitations of single-network materials. The stiff first network fractures under stress, dissipating mechanical energy and preventing catastrophic failure, while the flexible second network maintains structural integrity. This architecture enables simultaneous high strength (tensile strength >1 MPa) and high stretchability (elongation at break >400%), properties rarely achieved in conventional materials.

##### Elastomer-Conductive Filler Composites

Elastomers and self-healing polymers—including styrene–ethylene–butylene–styrene (SEBS), polyurethane, and polydimethylsiloxane (PDMS)—can be combined with conductive fillers such as graphene, carbon nanotubes, silver nanoparticles, and liquid metal to form hydrogel composites with enhanced stretchability, conductivity, and biocompatibility [175]. Liquid metal, commonly used for its superior thermal and electrical conductivity (approximately 10^6^ S/m) and excellent mechanical properties, creates percolating conductive pathways within elastomeric matrices [176]. The inherent flexibility of elastomeric backbones, combined with the high conductivity of liquid metal, enables wearable devices that stretch to over 400% strain while maintaining electrical functionality, critical for applications requiring conformability to curved body surfaces and dynamic motion.

Thus, composite hydrogels demonstrate superior performance compared to pure natural polymer hydrogels by maintaining aqueous biocompatibility while incorporating mechanical robustness and electrical functionality. Cellulose nanofibers can create reinforcing networks, enhancing tensile strength and elastic modulus without compromising inherent flexibility. Graphene oxide integration can improve both mechanical properties and electrical conductivity by forming a percolation network, enabling multifunctional sensing capabilities. These composites achieve stretchability exceeding 400% strain while maintaining electrical conductivity in the range of 0.01–0.1 S/m [159], making them suitable for simultaneous mechanical and electrochemical sensing applications. Further enhancement through the incorporation of inorganic particles such as clay nanoparticles, silica, or metal oxides reinforces the polymer network while imparting additional functionalities—silver nanoparticles provide antibacterial properties, while metal oxides improve thermal conductivity for temperature-responsive applications. These composite platforms enable spatially controlled mechanical properties and stimuli-responsive behaviour through multi-layered architectures, in which different layers are optimized for specific wearable devices and textile functions.

##### Integrating Functionality for Wearable Applications

Composite hydrogels address the multifunctional requirements of modern wearable devices by simultaneously providing mechanical flexibility, electrical conductivity for sensing and power delivery, biocompatibility for skin contact, self-healing capabilities for durability, and responsiveness to environmental stimuli, including temperature, humidity, and mechanical deformation. The strategic layering of different composite materials enables wearable devices to integrate multiple functionalities—strain sensing via piezoresistive layers, thermal sensing via thermochromic or thermoelectric layers, and biomarker detection via electrochemical sensing layers—all within a single conformal skin-contact device. The combination of natural polymer biocompatibility, synthetic polymer tunability, and nanoparticle functionality creates materials that balance performance requirements with biological safety and environmental sustainability, making them ideal platforms for next-generation wearable devices and textiles.

## 4. Hydrogel-Based Smart Wearable Devices and Textiles

Hydrogels are becoming increasingly important in intelligent wearable devices and textiles because they offer soft, biocompatible, and ionically or electronically conductive interfaces—capabilities that rigid or purely elastomeric materials cannot provide. However, current systems face significant limitations, including dehydration, mechanical fatigue, low energy density in energy storage applications, and difficulties in achieving reliable large-scale integration with electrodes and fabrics. Research reviewed in this section demonstrates that rational control of hydrogel composition and crosslinking—through strategies such as double and triple networks, nanocomposite architectures, tailored water states, and balanced ionic versus electronic conduction—can substantially enhance performance in batteries, supercapacitors, sensors, nanogenerators, and hydrogel–textile hybrids.

To illustrate these connections, Table 2 summarizes representative hydrogel-based devices across smart batteries, supercapacitors, nanogenerators, textiles, and multifunctional hybrids, while Table 3 details the material compositions and crosslinking mechanisms that underpin their properties. Furthermore, recent patented innovations involving hydrogels in wearable sensors, electrodes, smart fabrics, and energy systems (Table 4) highlight growing translational interest and underscore the role of hydrogels in improving comfort, performance, and sustainability. Finally, this section explores how artificial intelligence (AI) and the Internet of Things (IoT) are being integrated with hydrogel-based platforms to optimize design, enable real-time monitoring and data analytics, and expand device functionality.

### 4.1. Smart Batteries

Hydrogels play versatile roles in smart batteries, functioning as electrolytes, electrodes, binders, separators, and even functional interlayers, as highlighted in recent reviews [2,31,158]. Their unique properties make them highly suitable for flexible and wearable energy systems. Key advantages include their high water content and superabsorbency, which enable excellent ionic conductivity; a crosslinked 3D porous network that provides continuous ion pathways and mechanical robustness; controllable swelling and volume changes that allow adaptation to mechanical and thermal stress; and abundant functional groups that can coordinate metal ions, suppress side reactions, and mitigate dendrite growth [2,31,177].

Recent developments in lean-water and “water-structured” hydrogels have further expanded the electrochemical stability window and improved ion stabilization. These advances bring hydrogel electrolytes closer to the target ionic conductivity range of 0.01–0.1 S/cm and operational temperature windows relevant to wearable applications (approximately −20 to 60 °C). The 3D porous structure of hydrogels also helps homogenize ion flux and reduce local current hotspots, thereby suppressing metal dendrite growth and improving cycling stability.

In addition, immobilizing liquid electrolytes within the hydrogel framework reduces leakage and mitigates dissolution of active materials, supporting cycle lifetimes exceeding 1000 cycles and capacity retention above 90%, which are essential for reliable wearable devices. Functional groups such as carboxyl (–COOH) and sulfonate (–SO_3_^−^) enhance ion confinement, promote uniform metal deposition, and regulate interfacial reactions, particularly at zinc and other metal anodes [31].

Collectively, these design principles guide the development of hydrogel-based electrolytes that narrow the gap toward target performance metrics for flexible batteries—such as energy density above 250 Wh/kg and high-capacity retention under deformation—while maintaining softness, safety, and adaptability.

**Figure 3:**
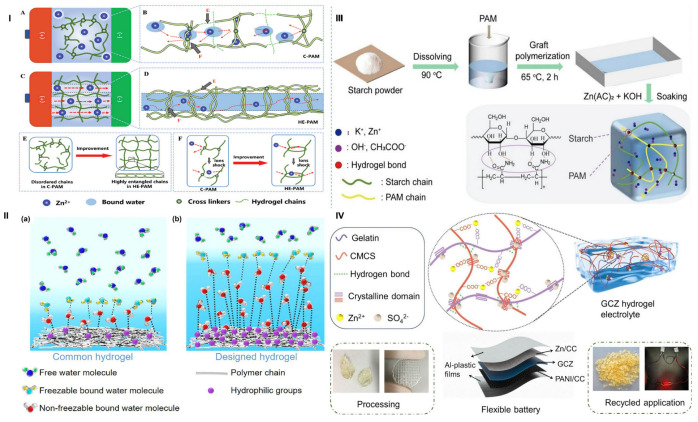
Illustration of (**I**) C-PAM and HE-PAM hydrogel electrolytes. The internal structure of (**A**,**B**) C-PAM and (**C**,**D**) HE-PAM hydrogel electrolytes. The improvements from the C-PAM hydrogel to the high-quality HE-PAM: (**E**) The disordered chain structure in C-PAM versus the long and stretchable chains in HE-PAM. (**F**) The crosslinking chains in C-PAM versus the highly entangled chains in HE-PAM. Reproduced with permission from [178]. Copyright 2024, John Wiley and Sons. (**II**) The three different states of water in hydrogels. (**a**) Common hydrogels and (**b**) the designed supramolecular hydrogels with tuned water states. Reproduced from [179]. Copyright 2024, American Chemical Society. (**III**) Synthesis of the S-PAM hydrogel electrolyte. Reproduced with permission from [181]. Copyright 2024, Elsevier. (**IV**) GCZ-x hydrogel electrolyte integrating the Hofmeister effect and multiple non-covalent interactions. Reproduced with permission from [183]. Copyright 2024, Elsevier.

Shen et al. [178] designed a highly entangled polyacrylamide (HE-PAM) hydrogel electrolyte for Zn/MnO_2_ batteries, focusing on optimizing chain entanglement and water state to improve both ion transport and mechanical stability (Figure 3I). By increasing monomer content (longer chains and more durable networks), reducing crosslinker content (fewer rigid junctions and less Zn^2+^ rebound), and promoting a continuous free-water layer rather than bound water, HE-PAM fundamentally altered ion transport in the hydrogel framework, enabling fast Zn^2+^ migration. The resulting electrolyte achieved ionic conductivity of 0.0225 S/cm and an elastic modulus of 166 kPa and could be stored for six months while withstanding high current shocks without structural collapse. In Zn/MnO_2_ batteries, HE-PAM provided an electrochemical window of 0.5–2.0 V and maintained 80.05% capacity after 2000 cycles at 10 A/g, with fast charge–discharge capability up to 35 A/g. These metrics are still below the energy density targets for flexible Li-ion cells but are highly competitive for aqueous Zn-based wearables, approaching the Table 1 target of >90% capacity retention over >1000 cycles under mechanical stress.

Li et al. [179] pursued a complementary strategy by tuning the water states in a supramolecular hydrogel electrolyte to maximize non-freezable bound water and minimize free water. The strategies employed involved carefully selecting monomers with various hydrophilic groups (i.e., –OH, –CONH_2_) to enhance the hydrophilicity of the polymer chains and incorporating zwitterionic polymers as super-hydrophilic units (i.e., –NH_4_^+^, –SO_3_^−^) (Figure 3II). Using PVA, glucose, zwitterionic poly(sulfobetaine methacrylate), acrylamide copolymers, sodium alginate, and laponite, they created a hydrogel with >0.4 mg/mg non-freezable bound water and <0.5 mg/mg free water, which improved low-temperature stability, suppressed side reactions, and widened the voltage window. In aqueous K-ion batteries, this electrolyte delivered a high cell voltage (~1.9 V) and stable cycling over >3000 cycles. Although the absolute energy density is still below rigid Li-ion benchmarks (250–300 Wh/kg), the cycle life and stability under aqueous conditions compare favorably with Table 1 targets for flexible systems, particularly in terms of long-term cycling and safe operation.

Other studies have focused on polyanionic hydrogel frameworks to guide metal-ion transport. Cong et al. [180] developed poly(2-acrylamido-2-methyl-1-propanesulfonate) zinc hydrogels in which Zn^2+^ migrates along a confined polyanionic “tunnel,” leading to uniform zinc deposition and suppressed dendrite formation. These conductivities approach the lower bound of the 0.01 S/cm target window in Table 1, and the high transference numbers align with the need for efficient ion transport and minimal polarization.

To enhance sustainability and mechanical robustness, many recent works incorporate natural polymers. Dai et al. [181] synthesized a starch-reinforced polyacrylamide (S-PAM) hydrogel electrolyte for flexible zinc–air batteries (FZABs) (Figure 3III). The S-PAM electrolyte exhibited high ionic conductivity (113 mS/cm) and a discharge voltage of ~1.3 V, supporting continuous operation for >40 h with stable output under bending and impact. An FZAB using S-PAM and a CoFe–N–C catalyst achieved a power density of 30 mW/cm^2^ and a specific capacity of 694 mAh/g. These values approach or exceed the supercapacitor-level targets in Table 1 for power density, though they still lag behind Li-based flexible batteries in terms of specific energy. Nonetheless, they demonstrate that hydrogel-based zinc–air systems can deliver practical power and capacity for many wearable applications, especially when high safety and breathability are prioritized over maximum Wh/kg.

Li et al. [182] prepared a carboxymethyl cellulose/polyacrylamide (CMC/PAM) double-network hydrogel electrolyte for flexible yarn zinc-ion batteries, achieving tensile deformation of 2700% and ionic conductivity of 0.0637 S/cm. Devices fabricated with this electrolyte exhibited an areal capacitance of 170.23 μAh/cm^2^ at 1 mA/cm^2^ and retained 73.14% capacitance after 100 cycles under deformation. While the cycle number is still below the 1000-cycle target in Table 1, the combination of high stretchability and moderate conductivity demonstrates progress toward mechanically resilient, fiber-shaped power sources compatible with textiles.

Ji et al. [183] used carboxymethyl chitosan and gelatin to create GCZ-x hydrogels with multiple non-covalent interactions and Hofmeister effects, yielding recyclability (>80%), tunable stiffness and toughness, and dual temperature/pH responsiveness (Figure 3IV). A Zn-ion battery using GCZ-x as the electrolyte delivered high specific capacity and excellent cycling performance (2200 h at 0.1 A/g) without zinc dendrite formation. This exceptional cycle life exceeds the Table 1 targets for flexible batteries and highlights how carefully engineered hydrogel networks can provide long-term stability that rivals or surpasses rigid systems, although energy density still depends on electrode chemistries.

Fu et al. [184] fabricated a hydrogel structure rich in -COOH groups crosslinked by polyvinyl alcohol and xanthan gum, having excellent ionic conductivity (18.86 mS/cm) and zinc-ion migration number (0.8), limiting side reactions and hydrogen evolution reactions, and beneficial to uniform deposition of Zn^2+^, achieving stable long cycling life in Zn/Zn symmetrical battery. In an effort to meet sustainability demands, researchers have recently incorporated sustainable natural polymers (e.g., cellulose, chitin, and their derivatives) into their hydrogel designs [181,182,183,185,186].

Hydrogel-based smart batteries rely not only on composition but also on tailored crosslinking mechanisms that balance ionic transport, mechanical robustness, and environmental stability. Common strategies include chemical covalent crosslinking (e.g., N,N′-methylene bisacrylamide in PAM and PAMPS-type networks) to provide permanent 3D frameworks with defined pore structures; physical crosslinking through hydrogen bonding (PVA–cellulose nanofibers, starch–PAM) and chain entanglement (HE-PAM) to dissipate mechanical energy and improve toughness; and ionic coordination (Zn^2+^–carboxylate in CNF, alginate, or carboxymethyl chitosan systems) to add sacrificial, reversible bonds that enhance self-recovery and suppress dendrite formation [178,179,184]. Double- and triple-network architectures integrate these mechanisms—stiff covalent or ionically crosslinked networks for load-bearing, combined with softer, physically crosslinked networks for stretchability—yielding hydrogels that maintain high ionic conductivity while withstanding large strains and repeated cycling, which is critical for wearable batteries that undergo continuous deformation.

Overall, current hydrogel-based battery systems approach or meet the target ranges in Table 1 for ionic conductivity, cycling stability, and safe operation, especially for aqueous Zn-based systems and zinc–air chemistries. However, they generally remain below the energy density targets set for flexible Li-ion batteries (current 100–200 Wh/kg, target >250 Wh/kg) and should continue to improve mechanical durability under high strain and long-term environmental exposure to fully match rigid counterparts in demanding wearable scenarios. Market analyses indicate that the global industrial battery sector (including advanced chemistries relevant to smart and wearable batteries) is projected to grow from US$23.19 billion in 2024 to US$39.78 billion by 2031 (CAGR ≈ 8%), while proxy segments such as hydrogel-electrolyte-based electronic skins and patches are expected to grow at 14–15% CAGR through 2032, underscoring strong commercial pull for thin, flexible, and safe power sources [187,188,189].

Hydrogels thus play a central role in enabling thin, flexible, cut-resistant, and more sustainable batteries that can be seamlessly integrated into electronic skins, patches, and textiles. The convergence of advanced crosslinking designs (covalent, physical, and ionic), natural polymer incorporation, and green processing with a rapidly expanding smart-battery market suggests that hydrogel-enabled systems will be key to meeting performance, safety, and sustainability targets in next-generation wearables, particularly when coupled with ongoing improvements in electrode materials and device encapsulation.

### 4.2. Smart Supercapacitors

Supercapacitors bridge the gap between batteries and conventional capacitors by offering much higher power density than batteries and significantly greater energy density than traditional capacitors. They are generally classified into two types: electric double-layer capacitors (EDLCs), which store charge through ion adsorption at the electrode–electrolyte interface, and pseudocapacitors, which rely on fast, reversible redox reactions on the electrode surface to provide additional charge storage. Their overall performance depends on the combined behaviour of electrodes, electrolytes, separators, and current collectors. Conventional supercapacitors are typically rigid, limiting their stretchability and compressibility for wearable applications. Flexible supercapacitors aim to overcome these limitations and, in comparison with the target values in Table 1, should deliver high power density (>5 kW/kg), reasonable energy density (tens of Wh/kg), long cycle life (>5000–10,000 cycles), and stable performance under mechanical deformation, while remaining thin and conformable [190,191].

Hydrogel electrolytes are central to many flexible and wearable supercapacitor designs because they combine high ionic conductivity, water retention, and mechanical deformability with biocompatibility and, in some cases, biodegradability [192,193]. Their water-rich networks dissolve salts to provide efficient ion transport pathways and can be engineered for additional functionalities such as stretchability, compressibility, anti-freezing behaviour, self-healing, electrochromism, and shape memory [124,125,192,193,194,195,196,197]. Compared with traditional liquid or solid polymer electrolytes, hydrogel-based systems more easily meet the mechanical requirements for wearables—such as large strain tolerance and repeated bending—while approaching or achieving the ionic conductivity range (0.001–0.1 S/cm) and cycle-life targets outlined in Table 1.

Despite these advantages, hydrogel-based supercapacitors still typically lag behind rigid devices in terms of maximum energy density and upper operating voltage window, particularly at high voltages (>2.5–3 V). Addressing these limitations remains a key focus for advancing hydrogel-based supercapacitor technology for next-generation wearable applications.

Wang et al. [197] demonstrated an early hydrogel-based flexible supercapacitor using a PANI/PVA-hydrogel/PANI structure, where polyaniline was polymerized on a crosslinked PVA hydrogel electrolyte prepared by covalent crosslinking and film casting. The device achieved an areal capacitance of 488 mF/cm^2^ and showed only slight capacitance degradation after 1000 bending cycles, illustrating that hydrogel electrolytes can provide both high capacitance and mechanical robustness. Although the energy density was not explicitly reported, the areal capacitance and stable performance under bending were competitive for early flexible devices but below the long-cycle (>5000 cycles) and high-power targets of modern systems. This work established a baseline for hydrogel-based EDLCs: good mechanical resilience and acceptable capacitance, but still modest voltage and energy density compared with the target ranges in Table 1.

**Figure 4:**
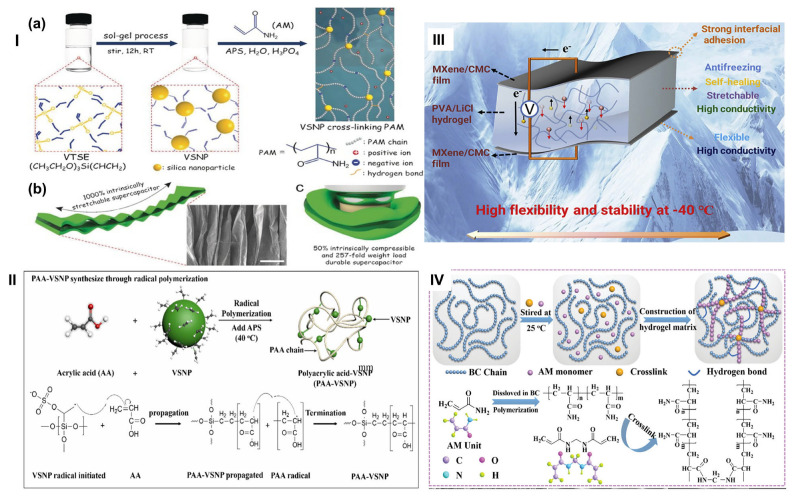
Schematic illustration of (**I**) (**a**) VSNPs preparation from VTES followed by preparation of the VSNPs–PAM electrolyte from VSNPs (crosslinker), acrylamide (AM, main monomer), ammonium persulfate (APS, initiator) and phosphoric acid (proton source). (**b**) The intrinsically 1000% stretchable supercapacitor and an SEM image of the wavy PPy@CNT paper electrode. Scale bar: 1 mm. (**c**) The 50% compressible supercapacitor holding a 257-fold weight load. Reproduced with permission from [195]. Copyright 2017, John Wiley and Sons. (**II**) The synthesis of PAA-VSNP. Reproduced with permission from [163]. Copyright 2023, Elsevier. (**III**) The flexible and wearable supercapacitors were assembled by sandwiching PVA/LiCl hydrogel electrolyte with two MXene/CMC film electrodes. Reproduced with permission from [125]. Copyright 2022, Elsevier. (**IV**) The synthesis process for the BC/PAM hydrogel via a simple polymerization approach. Reproduced with permission [198]. Copyright 2021, John Wiley and Sons.

Huang et al. [195] advanced this concept by synthesizing a polyelectrolyte hydrogel composed of proton-incorporated PAM crosslinked with vinyl hybrid silica nanoparticles (VSNPs) (Figure 4II). PAM provided toughness, while VSNPs served as stress buffers to dissipate energy under large strain. The VSNPs–PAM hydrogel exhibited stretchability up to 1500% without visible cracking and, when combined with PPy-electrodeposited CNT paper electrodes, enabled a supercapacitor that was 1000% stretchable and 50% compressible. The device maintained 99.4% capacitance retention after 260% stretching and 50% compression, demonstrating outstanding mechanical stability. While detailed energy/power metrics were not emphasized, the device clearly exceeded typical mechanical targets (elongation > 400%) and approached or surpassed the Table 1 target of >90% capacitance retention over thousands of mechanical cycles, albeit still with relatively modest energy density at low operating voltage.

Kim et al. [196] developed a poly(acrylic acid)–vinyl-functionalized silica nanoparticle (PAA–VSNP) hydrogel electrolyte using sol–gel and radical polymerization for a flexible sodium-ion hybrid supercapacitor (SHSC) (Figure 4I). The PAA–VSNP hydrogel exhibited high stretchability (up to 1122.75% strain), ionic conductivity of 20.23 mS/cm (0.02023 S/cm), and low activation energy (0.106 eV), attributes that support fast Na^+^ transport. When integrated between activated carbon (AC) and Na_3_V_2_(PO_4_)_3_/carbon composite (NVP@C) in an AC//PAA–VSNP//NVP@C SHSC, the device delivered an energy density of 86.95 Wh/kg and a power density of 19.94 kW/kg, with stable operation over 4000 cycles. These values exceed the power density target for supercapacitors in Table 1 (>5 kW/kg) and provide energy density comparable to or higher than many flexible supercapacitors, approaching the lower end of flexible battery-level energy (though still below high-performance Li-ion cells). The ionic conductivity lies comfortably within the 0.001–0.1 S/cm range, and the cycle life (4000 cycles) is close to but slightly below ambitious targets (>5000–10,000 cycles) for long-lived wearable supercapacitors.

Je et al. [194] addressed the limitations of hydrogel electrolytes under harsh conditions, such as low temperature and high voltage, by designing a triple-network hydrogel polymer electrolyte (TNPE) through one-step thermal radical polymerization. The TNPE consisted of lithium acrylate (LiA) as a hydrophilic monomer, two hydrophobic monomers (LMA and BMA), and LiCl salt, crosslinked via (1) covalent bonds (EGDMA, MBAA), (2) hydrophobic associations (alkyl side chains), and (3) ionic coordination (LiCl and LiA species). This triple crosslinking strategy yielded a hydrogel with ionic conductivity of 0.0056 S/cm at −30 °C, high water retention (80% after 20 days), and stable mechanical properties at sub-zero temperatures. A TNPE-based all-solid-state supercapacitor with activated carbon electrodes operated stably at 2.25 V, with specific capacitance of 24 F/g, energy density of 17 Wh/kg, and power density of 671 W/kg, retaining ≈100% capacitance after 10,000 cycles and maintaining performance under bending and folding. Compared with Table 1, the TNPE system clearly meets or surpasses the cycle-life target (>10,000 cycles) and demonstrates robust low-temperature operation with adequate power density, although the energy density remains moderate relative to high-energy flexible devices.

To integrate self-healing and electrochromism, Guo et al. [124] developed a PVA-based self-healing hydrogel electrolyte paired with Au-coated PANI/tungsten oxide nanowire electrodes, forming an electrochromic symmetrical supercapacitor. The device exhibited an areal capacitance of 61 mF/cm^2^ and retained 85% of its rate capacity as current density increased from 0.1 to 4 mA/cm^2^. The supercapacitor changed colour from lime green to dark blue as the applied voltage increased from 0.4 to 0.9 V vs. Ag/AgCl, providing visual feedback on charge state. After nine cutting–healing cycles, the capacitor recovered its mechanical integrity and capacitance without external stimuli, a result attributed to abundant hydrogen bonding between −OH groups in the PVA network. While the energy density and operating voltage are lower than those of high-performance SHSCs, the combination of self-healing, electrochromism, and flexible operation represents a different design priority: robust, user-interactive devices for low-voltage wearable applications, rather than maximizing Wh/kg.

Yin et al. [125] designed a PVA/LiCl hydrogel electrolyte with freeze–thaw processing and integrated it with MXene/carboxymethyl cellulose (CMC) film electrodes to fabricate a flexible, multifunctional supercapacitor (Figure 4III). LiCl disrupted hydrogen bonding between PVA chains, reducing crosslink density and enhancing ion transport, stretchability, and anti-freezing properties. The PVA/LiCl hydrogel showed skin-like softness, self-adhesion, self-healing, and high ionic conductivity, while the MXene/CMC electrode offered flexibility (12.7 MPa at 5.9% strain) and high conductivity (267 S/m). The assembled device exhibited a specific capacitance of 113.13 mF/cm^2^ and maintained ~95% capacitance under mechanical deformation and at −40 °C, demonstrating strong anti-freezing and mechanical resilience. In relation to Table 1, the power and energy densities are moderate, but the mechanical and low-temperature performance clearly meet or exceed the targets for wearable operation in extreme environments.

Cui et al. [199] took a more sustainable approach by employing nitrogen-doped carbon dot/graphene composite hydrogels derived from corncob lignin as flexible supercapacitor electrodes. Physical crosslinking via π–π interactions and electrostatic forces anchored nitrogen-doped carbon dots onto graphene sheets, forming a stable 3D network that enhanced electrochemical performance. The resulting electrode delivered specific capacitance of 387 F/g at 1 A/g and retained 75.7% of its capacitance at 7 A/g, demonstrating good rate capability. An assembled lignin-based supercapacitor showed 92.3% capacitance retention after 5000 cycles and energy density of 25.6 Wh/kg at a power density of 243 W/kg, while remaining stable under bending. These values compare favourably with the Table 1 targets for supercapacitors (energy densities on the order of tens of Wh/kg, 5000+ cycles, and mechanical robustness), underscoring that biomass-derived hydrogels can meet performance requirements while improving sustainability.

Li et al. [198] reinforced PAM hydrogels with bacterial cellulose (BC) nanofibers to create a BC/PAM hydrogel electrolyte that combined high ionic conductivity (125 mS/cm, or 0.125 S/cm) with excellent elasticity (~1300% strain) and tensile strength (330 kPa) (Figure 4IV). BC’s ultrafine interconnected nanofiber network and abundant hydroxyl groups provided mechanical reinforcement, enhanced water retention, and preserved ionic pathways. When assembled with graphene-encapsulated polyester fibers loaded with PANI, the resulting all-solid-state supercapacitor exhibited high areal capacitance (564 mF/cm^2^), good rate capability, favourable energy and power densities, and minimal capacitance degradation after repeated bending. Compared with Table 1, the ionic conductivity exceeds typical target values for hydrogel electrolytes, and the cycle stability under deformation aligns well with wearable requirements, though the energy density still falls short of the very highest targets for advanced flexible storage.

Hydrogel-based electrolytes and electrodes thus enable flexible, durable supercapacitors that deliver rapid power, maintain performance under large strains, and can be integrated into garments, patches, and soft systems. Many of the systems described achieve or exceed Table 1 targets for power density and cycle life (often >5000–10,000 cycles) and meet or surpass ionic conductivity goals in the 0.001–0.1 S/cm range, particularly when using carefully engineered double- or triple-network hydrogels. However, most devices still operate at moderate voltages (typically ≤2.5 V) and exhibit energy densities that, while competitive with supercapacitors (up to ~25–87 Wh/kg), generally remain below those of flexible Li-ion batteries. The projected ~16% CAGR for flexible and wearable supercapacitors to 2032 [200] reflects strong market demand driven by smart textiles, self-powered wearables, and IoT nodes that require lightweight, conformable, and safe energy storage. This growth, together with advances in hydrogel–conducting polymer composites and eco-friendly materials, suggests that hydrogel-based supercapacitors will play a key role in meeting power, durability, and sustainability targets for next-generation wearable devices.

### 4.3. Smart Sensors

Compared with traditional elastic sensors that are often bulky, rigid, and poorly biocompatible, hydrogel-based smart sensors provide soft, conformal, and skin-friendly interfaces with high signal transduction efficiency. These characteristics make them particularly suitable for on-skin and in-textile human–machine interfaces. Hydrogel sensors offer several key advantages, including intrinsic mechanical adaptability to large strains and compression, strong and reversible adhesion, self-healing capability, anti-freezing behaviour, and high ionic or mixed ionic–electronic conductivity.

These features enable hydrogel sensors to meet or closely approach the performance targets for wearable sensing outlined earlier: large strain tolerance (>200–400%), stable operation across a wide temperature range (−20 to 60 °C), conductivity in the 0.001–1 S/m range, and robust performance over hundreds to thousands of deformation cycles. In contrast, conventional hydrogel-free or rigid sensors often struggle to maintain comfort, reliability, and signal stability during motion, limiting their suitability for next-generation wearable applications.

**Figure 5:**
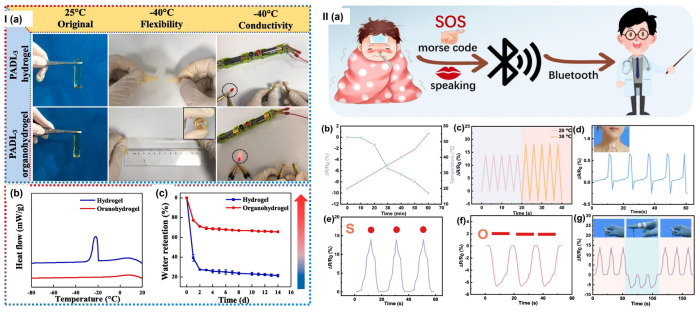
(**I**) PADL3 hydrogel and organohydrogel (**a**) anti-freezing properties at −40 °C for 24 h. (**b**) DSC curves. (**c**) Water retention after being placed at room temperature. Reproduced with permission from [201]. Copyright 2024, Elsevier. (**II**) Sensor performance of NaCl/SA/PAM/PNIPAM/CaCO3 hydrogel (**a**) patient communicating with doctor through finger motions and vocalization, (**b**) resistance curve at different temperatures, (**c**) resistance curve of 50% strain after stretching-recovery at different temperatures, (**d**) resistance curves monitoring on throat, (**e**,**f**) translation of electric signals into Morse Code, and (**g**) language identification in response to finger motions. Reproduced with permission from [135]. Copyright 2021, Elsevier.

Jiang et al. [201] designed multifunctional, highly stretchable and conductive PADL organohydrogels based on lignosulfonate nanoparticles (nano-LGS) doped poly(acrylic acid-co-2-(methacryloyloxy)ethyl trimethylammonium chloride) in a glycerol/H_2_O solvent, using a one-step in situ polymerization process (Figure 5I). The material combines anionic acrylic acid (rich in –COOH) and cationic DMC to form a polyampholyte network with strong electrostatic interactions and intrinsic adhesiveness, while glycerol–water binary solvent extends the anti-freezing range and improves water retention. Lignosulfonate nanoparticles provide a high surface area and abundant aromatic and sulfonic groups, reinforcing mechanical strength, conductivity, and antibacterial behaviour [201,202,203]. The resulting organohydrogel showed strong adhesion, anti-freezing capability down to −40 °C, self-healing, and sufficient conductivity to function as a strain sensor with a gauge factor (GF) of 7.91 for monitoring body motion and handwriting. In comparison with the sensor targets drawn from Table 1 (strain sensing with GFs of ~4–10, stability across sub-zero temperatures, and durable operation under repeated deformation), the PADL organohydrogel system performs competitively, although long-term cycling data and detailed noise/stability metrics remain less fully quantified.

Wu et al. [204] further exploited multi-component design by using freeze–thaw crosslinking between polyvinyl alcohol (PVA) and aramid nanofibers (ANF), a tannic acid (TA)/CaCl_2_ mixture as a crosslinker, and a DMSO/water co-solvent to fabricate PAT5/CaCl_2_-5% (DMSO/H_2_O) ion-conductive hydrogels. The combined covalent/physical crosslinking (PVA–ANF network), hydrogen bonding and coordination bonds (TA with Ca^2+^), and DMSO-modulated water structure produced a hydrogel with high elongation at break (~755%), tensile strength of 6.25 MPa, conductivity of 3.09 S/m, anti-freezing performance down to −41.7 °C, good moisture retention, and antibacterial activity. As a strain sensor, PAT5/CaCl_2_-5% showed GFs of 1.02 (<100% strain) and 0.93 (100–200% strain) for real-time monitoring of finger, knee, and elbow bending as well as speech-related motions. The conductivity exceeds the 0.01–0.1 S/m target range, and the temperature range surpasses typical wearable requirements, but the sensitivity is moderate compared with high-GF systems; this highlights a familiar trade-off between very high stretchability and ultrahigh sensitivity, which subsequent studies attempt to balance.

Wang et al. [135] developed a wide-temperature, highly conductive polyelectrolyte hydrogel (NaCl/SA/PAM/PNIPAM/CaCO_3_) using sodium alginate (SA), acrylamide (AM), N-isopropylacrylamide (NIPAM), NaCl, Ca^2+^, and CaCO_3_ nanoparticles via a one-pot free-radical polymerization (Figure 5II). A dual network of PAM–PNIPAM covalent bonds is reinforced by hydrogen bonding with SA, while Ca^2+^/CaCO_3_ provides dynamic metal coordination sites that underpin self-healing. NaCl interacts with SA via electrostatic screening and with –COO^−^ groups, enhancing water binding, anti-freezing behaviour, and ionic conductivity. The resulting hydrogel exhibits conductivity of 2.75 S/m from −15 to 50 °C, elongation of 950% at stress ~24.3 kPa, fracture strain >2000%, and partial self-healing (~40% stretch length recovery). As a sensor, it shows high sensitivity (GF = 8.76 under large strain), enabling detection of finger motions, throat vibrations, and vocalization patterns, including translating output into Morse code and language identification. In relation to Table 1–type targets, this system satisfies or exceeds the mechanical requirements (elongation well above 400%) and offers conductivity at the upper end of desirable ranges for ion-conductive skins, with a GF similar to or better than many flexible strain sensors, though long-term cycling data beyond the reported tests would be needed to fully match the durability benchmarks (>1000 cycles).

Zhao et al. [126] explored a hybrid organic hydrogel combining MXene (Ti_3_C_2_T_x_), PEDOT:PSS, PVA, and polydopamine (PDA) to achieve a conductive, self-healing, and biocompatible sensor platform for ultra-sensitive human–computer interaction. In this design, PVA provides a flexible, biocompatible backbone; MXene and PEDOT:PSS form percolating electronic pathways with high conductivity via π–π stacking and electrostatic interactions; and PDA improves adhesion, mechanical strength, and durability. Multiple dynamic noncovalent interactions (hydrogen bonding between PVA and PDA, π–π interactions, and electrostatic interactions among MXene and PEDOT:PSS) enable rapid self-healing of both the mechanical structure and the conductive networks, with self-healing times as short as 1.8 s and conductivity of ~1.2 S/m. Sensors based on this hydrogel accurately captured finger and wrist bending, facial expressions, blinking, handwriting, and motion speed. Relative to benchmark expectations (conductivity ~0.001–1 S/m, fast response, high cyclic stability), this system sits at the high-conductivity end and demonstrates exemplary multifunctional sensing and self-healing, though detailed long-term cycling under sweat and environmental stress remains an open challenge.

Yu et al. [205] targeted extreme environmental adaptability by combining a PAM network with catechol-modified hyaluronic acid (HA-CA) and laponite nanoparticles to produce a temperature-tolerant, stretchable organohydrogel for epidermal sensors. Dual physical/chemical crosslinking endowed the hydrogel with super-stretchability (>8000%), strong, reversible adhesion, fast self-healing (<10 s), and conductivity of ~63 mS/m (0.063 S/m) even at −0 °C, along with good biocompatibility. When used as a strain sensor, it exhibited a broad sensing range (0–300% strain) and very high sensitivity (GF = 8.38–133.94 depending on strain regime), enabling discrimination of diverse human movements at low temperature. Compared with target values, this device surpasses typical stretch and sensitivity requirements, while its conductivity and low-temperature performance fall within desirable ranges. The main gap lies in the need for long-duration stability data under real-life wear, but the design clearly shows how organohydrogels can exceed conventional sensors in mechanical adaptability and sensitivity.

Wang et al. [136] synthesized a PMAA@MXene hydrogel sensor by polymerizing methacrylic acid with trisodium citrate dihydrate as a multifunctional crosslinker and MXene as a conductive filler. Hydrogen bonds between –COO^−^/–OH groups in the polymer and surface groups on MXene promoted homogeneous MXene dispersion and built a mechanically robust network. The resulting hydrogel exhibited elongation at break of 40.4%, toughness of 110 kJ/m^3^, and a tunable Young’s modulus of 23.5–92 kPa—similar to soft tissue—enabling comfortable skin-like interfaces. The PMAA@MXene sensor showed a GF of 4.41 in tension, durability over 1000 loading–unloading cycles, and high accuracy (~0.97) in handwriting recognition tasks. While its stretchability is more modest than that of some ultra-stretchable designs, its modulus alignment with human tissue and its stable performance over 1000 cycles meet key mechanical and durability targets for many wearable applications.

Across these studies, a few technical themes emerge. First, multi-network and composite designs (organohydrogels, double/triple networks) are crucial for reconciling high stretchability, toughness, and conductivity—addressing a gap that single-network hydrogels rarely close. Second, combinations of ionic conduction (Na^+^, Li^+^, Ca^2+^-based hydrogels) and electronic conduction (MXene, PEDOT:PSS, PPy, CNTs) enable sensors to achieve or exceed the conductivity required for low-noise signal acquisition while remaining soft and conformal. Third, solvent engineering (glycerol/water, DMSO/water) and salt selection (NaCl, LiCl) are key to achieving anti-freezing and moisture-retentive behaviour, ensuring stable operation from sub-zero temperatures up to typical skin or ambient temperatures. Finally, dynamic supramolecular and coordination bonds (hydrogen bonding, metal–ligand coordination) underpin rapid, repeatable self-healing, helping devices maintain performance after mechanical damage and contributing to long-term reliability benchmarks that align with Table 1 style targets (hundreds to thousands of cycles under strain).

Functionally, hydrogel-based sensors now meet or surpass many application-level requirements for wearable monitoring: strain ranges from small facial movements to large joint bending, gauge factors from ~4 to >100 depending on design, operation down to −40 °C, and stable performance under repeated deformation. They are particularly well-suited for use in epidermal electrodes, multimodal motion and vocalization sensors, and textile-integrated sensing arrays. The projected 13.9–16.1% CAGR of hydrogel-enabled epidermal sensors and related wearable sensing platforms through 2032 [206,207] reflects strong commercial pull driven by digital health, sports performance tracking, and consumer wellness applications. As user expectations and regulations increasingly emphasize skin safety, comfort, and environmental responsibility, water-based and natural polymer hydrogels—combined with advanced nanocomposites and AI-enabled signal processing—are likely to remain at the core of next-generation smart sensing systems.

### 4.4. Smart Nanogenerators

Hydrogel-enabled piezoelectric nanogenerators (PENGs) and triboelectric nanogenerators (TENGs) are emerging as soft, self-powered platforms for motion energy harvesting and biomechanical sensing. Their tunable mechanical properties, strong adhesion, self-healing capability, and biocompatibility allow them to conform to skin and textiles, overcoming the limitations of rigid nanogenerators in stretchability, comfort, and safety [208,209,210,211,212,213,214,215,216,217,218,219]. Common hydrogel matrices include acrylamide, poly(acrylic acid), polyvinyl alcohol (PVA), and natural polymers such as cellulose, chitin derivatives, alginate, and gelatin. These are often combined with dielectric or piezoelectric fillers to enhance electrical output. Compared with the target values outlined in Table 1, hydrogel-based nanogenerators typically meet or exceed mechanical requirements for wearable devices—such as high strain tolerance and toughness—and generate voltages and currents sufficient for low-power electronics and sensing. However, long-term stability under conditions such as sweat exposure, dehydration, and high-frequency motion remains a significant challenge.

Crosslinking mechanisms play a critical role in enabling these hydrogels to satisfy concurrent requirements for mechanical robustness, elasticity, ionic transport, and interfacial stability with triboelectric or piezoelectric layers. Chemically crosslinked networks (e.g., BIS- or EGDMA-crosslinked acrylamide or acrylic acid systems) provide permanent 3D frameworks that maintain shape and mechanical integrity under repeated deformation. Physical crosslinks formed through hydrogen bonding (such as PVA–borax, PVA–cellulose, and PDA–polymer interactions) and hydrophobic associations introduce sacrificial bonds that dissipate energy and enable partial or full self-healing. Ionic and coordination bonds—such as Zn^2+^/Ca^2+^ linking cellulose or alginate chains, Fe^3+^–carboxylate coordination, and dynamic Schiff base (imine) linkages between oxidized polysaccharides and amines—create reversible crosslinks that reinforce the network while allowing stress relaxation, recyclability, and strong adhesion to diverse substrates.

These intertwined covalent, physical, and ionic crosslinking strategies underpin the double- and triple-network hydrogels used in TENGs and PENGs, enabling devices to operate under large strains, sub-zero temperatures, and repeated loading while maintaining stable electrical output. Continued optimization of these mechanisms is essential for improving durability and performance in real-world wearable applications.

**Figure 6:**
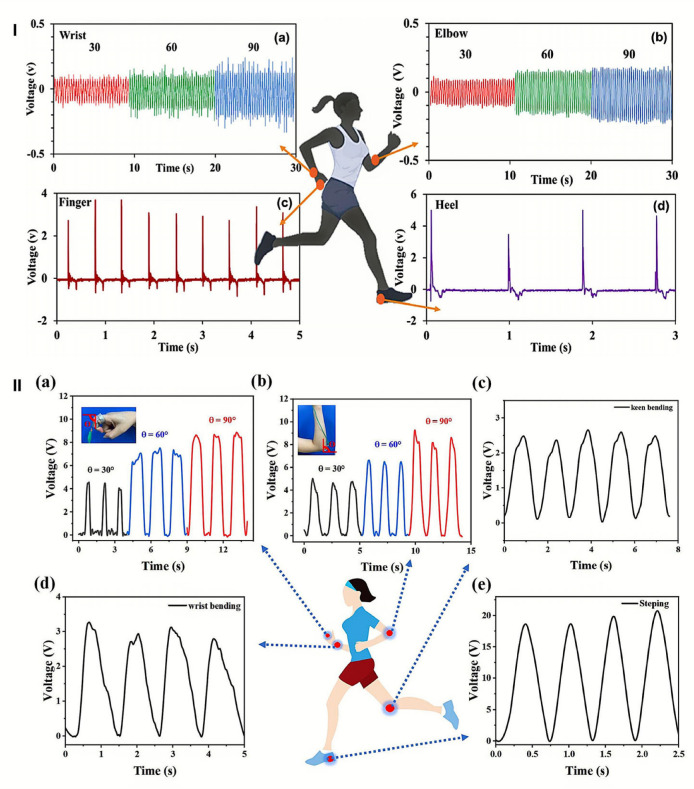
(**I**) The response of the optimized PVDF/SA-CCTOs-HA (PSCH5) composite piezoelectric hydrogel sensor’s output peak-to-peak voltage during different body motion gestures: (**a**,**b**) wrist and elbow bending angles, (**c**,**d**) finger and heel pressing and releasing motion. Reproduced with permission from [219]. Copyright 2024, Elsevier. (**II**) PTENG-based self-powered sensors developed for monitoring different human motions, flexural measurement and sensing different handwritten details. The self-powered sensor responds to voltage signals for (**a**) finger, (**b**) elbow joints with different degrees of flexion, (**c**) knee joint flexion, (**d**) wrist joint flexion, and (**e**) foot press. Reproduced from [215]. Copyright 2025, Elsevier.

Khazani et al. [219] prepared a piezoelectric hydrogel composite (PVDF/SA–CCTO–HA) via freeze-drying, combining poly(vinylidene fluoride) (PVDF) for mechanical strength, sodium alginate (SA) for biocompatibility, calcium copper titanate (CCTO) nanowires, and hydroxyapatite (HA) nanoparticles (Figure 6I). Freeze-drying created a porous network in which CCTO and HA enhanced dielectric properties, promoted β-phase formation in PVDF, and improved microstructure, piezoelectric behaviour, and mechanical performance compared with unmodified PVDF–SA. The optimized PSCH hydrogel (0.5% CCTO, 0.4% HA) achieved an open-circuit voltage of 7 V and short-circuit current of 3.5 μA—over fivefold higher than the base PVDF–SA hydrogel—alongside compressive strength of 8.2 MPa and tensile strength of 0.8 MPa. It reliably detected wrist, elbow, finger, and heel motions and could charge a 1 µF capacitor, illustrating both sensing and low-power energy storage capability. While direct comparison to Table 1 is indirect (since Table 1 focuses on storage rather than harvesting), the mechanical strength and deformation tolerance meet or exceed wearable requirements, and the output levels are sufficient for self-powered sensing and intermittent powering of ultra-low-power electronics.

Wang et al. [213] developed a double-network (DN) hydrogel for TENG applications by combining a rigid cellulose/Zn^2+^/Ca^2+^ network with a soft PVA–borax matrix. The rigid network provided stiffness and mechanical integrity through metal coordination and hydrogen bonding with cellulose, while the PVA–borax network contributed flexibility and additional hydrogen-bonded crosslinks. By tuning component ratios, they obtained tough, transparent, and self-adhesive hydrogels with tensile stress of 125.6–159.4 kPa, toughness of 265–541.5 kJ/m^3^, elongation up to 683.5%, and ~90.4% optical transmittance at 650 nm. The C3.5P3B2 formulation showed strong adhesion to diverse substrates (paper, foil, PDMS, plastics) and high ionic conductivity (0.4–2.5 S/m), with good anti-drying performance (only ~25–32% water loss after 30 days or under heat/low humidity). These properties allowed the hydrogel to act as a recyclable, transparent TENG electrode that could power LEDs (3 V) and function as a stylus while maintaining output after multiple recycling steps. Relative to target metrics, the conductivity significantly exceeds 0.01–0.1 S/m, mechanical stretchability is within or above desired ranges for wearables, and recyclability addresses durability and sustainability concerns not captured in traditional performance tables.

Long et al. [214] synthesized a PAAm–polydopamine (PDA) hydrogel for TENGs consisting of AM–BIS copolymers, self-polymerized PDA, and TMEDA as an accelerator. Covalent crosslinking by BIS established a PAAm network, while PDA introduced catechol groups that formed additional hydrogen bonds and π–π interactions with surrounding chains and triboelectric layers, imparting self-healing and strong adhesion. The hydrogel displayed excellent flexibility and self-healing (<10 min) and could stretch up to ~6000% strain. When used as a soft electrode with PTFE/VHB triboelectric layers in a single-electrode (SE) mode TENG, the PAAm–PDA hydrogel produced output voltages of ~230 V and currents of ~12 μA, outperforming conventional copper foil electrodes. Although power density and long-term cycling data were not fully detailed, the combination of gigantic stretchability, rapid self-healing, and higher electrical output illustrates how dynamic physical crosslinks (PDA-based) can deliver performance beyond rigid metal electrodes in deformable TENGs.

Hou et al. [215] fabricated BaTiO_3_-doped hydrogels based on oxidized sodium alginate, acrylic acid, acrylamide, and FeCl_3_, employed as electrodes in piezoelectric–triboelectric hybrid nanogenerators (PTENGs) (Figure 6II). The hydrogel network featured multiple crosslinking modes: dynamic Schiff base bonds between oxidized alginate and amines, Fe^3+^–carboxylate coordination, and extensive hydrogen bonding among polymer chains and BaTiO_3_ surfaces. This multi-crosslinking design yielded tensile stress of 2.14 MPa, tensile strain of 876%, toughness of 9.96 MJ/m^3^, conductivity of 0.14 S/m, and notable antimicrobial activity. The PTENG generated open-circuit voltages up to 222 V and short-circuit currents of 5.35 μA, enough to power commercial electronics and operate as a self-powered strain and tactile sensor. Mechanically, the device clearly meets or exceeds the deformation targets for wearable interfaces; electrically, the output levels and robustness under repeated motion suggest strong alignment with the requirements of continuous biomechanical energy harvesting and self-powered sensing.

Collectively, these studies show that hydrogel-enabled PENGs and TENGs can meet key mechanical and functional benchmarks for wearable systems: large stretchability (hundreds to thousands of percent), high toughness (kJ/m^3^ range), conductivity in or above the 0.001–1 S/m range, and stable electrical output under repeated deformation and environmental stress. While their “energy density” is not directly comparable to batteries or supercapacitors, their power output is sufficient for intermittent or continuous powering of low-power sensors, LEDs, and wireless nodes, especially when combined with energy storage elements. The projected 24–29.5% CAGR for hydrogel-based nanogenerators and related self-powered systems into the early 2030s [220,221] reflects strong demand from IoT, self-sustaining health devices, smart textiles, and battery-free electronics. As crosslinking strategies continue to evolve—optimizing combinations of covalent, physical, and ionic bonds—and as dehydration mitigation and textile integration improve, hydrogel-based TENGs and PENGs are poised to play a central role in distributed, autonomous power and sensing architectures in wearable and textile electronics.

### 4.5. Smart Fabrics

Hydrogels are increasingly being used to functionalize fabrics for applications such as energy harvesting and storage, motion and strain sensing, wireless communication, and multi-stimulus responsiveness, while maintaining or even enhancing comfort, breathability, and drape. In these hybrid systems, the fabric typically provides mechanical robustness, conformability, and washability, whereas the hydrogel contributes ionic or electronic conduction, adhesion, and stimulus-responsive behaviour.

Achieving strong hydrogel–fiber interfaces and long-term durability under bending, stretching, and laundering requires careful design of crosslinking mechanisms. Covalent bonds, physical interactions such as hydrogen bonding and polymer entanglement, and ionic or coordination bonds are central to ensuring tunable stiffness, adhesion, and structural integrity. These strategies enable smart fabrics to meet wearability and durability expectations while delivering performance targets similar to those outlined earlier for flexible batteries and supercapacitors—such as high strain tolerance, thousands of deformation cycles, and safe operation across a wide temperature range (−20 to 60 °C).

**Figure 7:**
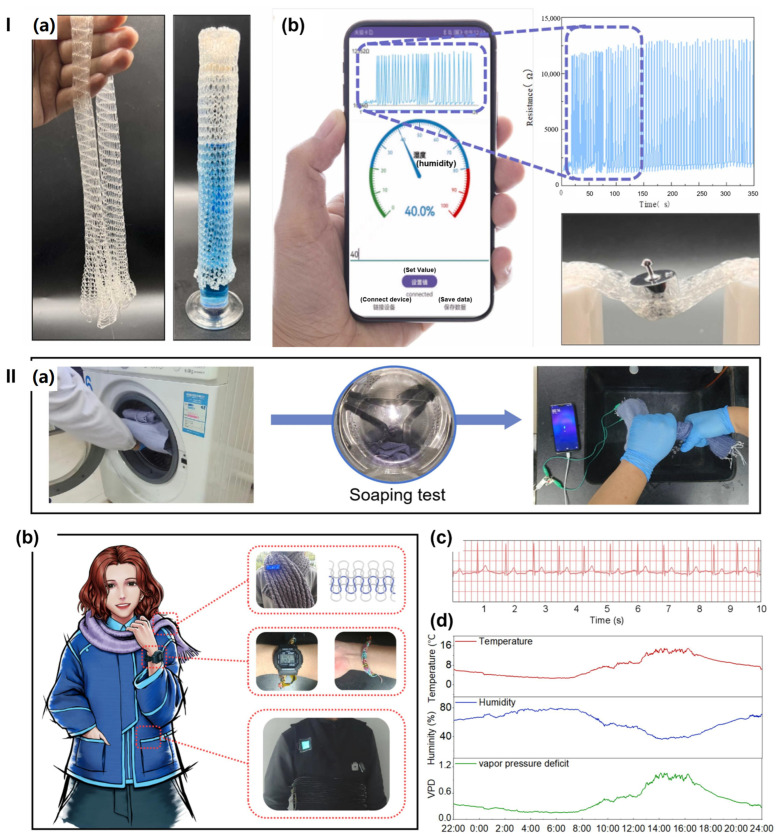
(**I**) Boric acid-crosslinked sodium alginate–zinc/guar gum (B-SZA/GG) hydrogel–fabric (**a**) digital image, (**b**) real-time monitoring system display. Reproduced with permission from [222]. Copyright 2025, Elsevier. (**II**) Smart cathode-less zinc–manganese (Zn–Mn) fiber-based battery: (**a**) soaping test using a washing machine and powering a mobile phone after washing, (**b**) demonstration of practical application of the fabric battery, (**c**) electrocardiography monitored and drawn using the HTS sensor in (**b**) with a running speed of 25 mm/s and a transmission gain of 10 mV/mm. (**d**) Environmental data, including temperature, humidity, and vapour pressure deficit, recorded by the HTS sensors in (**b**) and transmitted to the mobile app by Bluetooth. Reproduced with permission from [223]. Copyright 2024, Elsevier.

Guo et al. [222] developed boric-acid-crosslinked sodium alginate–zinc/guar gum (B–SZA/GG) hydrogel fibers via wet spinning (Figure 7I). Sodium alginate and guar gum form the polysaccharide backbone, while boric acid introduces dynamic borate–diol coordination crosslinks, and zinc ions in an ethylene glycol–water coagulation bath provide additional ionic crosslinking and freezing-point depression. By optimizing boric acid content and bath composition, fibers with 9% boric acid achieved tensile strength of 40.9 MPa, elongation of 233.1%, and a freezing point of −23.1 °C, plus strong antibacterial activity against *E. coli*. These hydrogel fibers exhibited high mechanical strength, antifreezing behaviour, water retention, and ionic conduction and were woven into fabrics integrated with electronics to build wireless sensor systems for real-time motion monitoring. Mechanically, the strength and elongation exceed typical textile targets for stretch garments, and the antifreeze performance and durability align well with Table 1 style requirements for operation in cold environments. However, the electrical performance is optimized for sensing rather than high-density energy storage.

Xu et al. [223] designed a high-safety, cathode-less Zn–Mn fiber battery to address the need for flexible, washable, and reliable power in smart fabrics. They employed a “reinforced-concrete” PTP composite hydrogel electrolyte by UV-curing a PAM-based precursor onto a thermoplastic polyurethane (TP) nanofiber membrane. The TP membrane, produced by coating polyacrylonitrile fibers with TPU nanofibers, provided a fibrous skeleton for mechanical reinforcement, while the PAM hydrogel supplied ionic pathways. Covalent crosslinking within the PAM network, physical entanglement with the TP scaffold, and hydrogen bonding between hydroxyl and amide groups created a robust yet flexible electrolyte that facilitated Zn^2+^ transport and suppressed hydrogen evolution and dendrite formation. A carefully engineered decoupled pH environment (weakly acidic near the cathode, neutral near the anode) further mitigated side reactions. This cathode-less configuration delivered a broad voltage plateau above 1.9 V, volumetric energy density of 227.08 mWh/cm^3^, capacity of 156.48 mAh/cm^3^, and 76.5% retention after 2700 cycles. These values compare favourably with the cycle and retention targets for flexible batteries in Table 1 and demonstrate that fabric-integrated Zn–Mn systems can approach or exceed conventional flexible Zn-based batteries in both safety and performance, even if overall Wh/kg still trails those of high-end flexible Li-ion cells. The fiber batteries remained functional after washing and cutting and were seamlessly integrated into commercial textiles as part of a health-tracking system (HTS) that combined sensors, charging units, and electroluminescent modules for real-time ECG and environmental monitoring (Figure 7II).

Ding et al. [224] developed a soft thermogalvanic hydrogel–fabric that converts body heat into electricity by weaving stretchable thermogalvanic fibers into fabric structures. Alternating p-type and n-type fiber units, each containing thermogalvanic redox couples in a hydrogel matrix, were woven directly into textiles without air-impermeable substrates, preserving breathability and comfort. The fabric adapted well to curved skin and continuous motion, generating open-circuit voltages of ~0.7 V in cold environments and up to 0.5 V during dynamic movements (e.g., at the elbow). Though overall power density was modest compared to advanced TENGs or supercapacitors, this approach demonstrated viable body-heat harvesting at the garment level with mechanically compliant hydrogel-based fibers.

Jiang et al. [225] integrated amorphous PVA hydrogels with elastic fabrics via coating and in situ freeze–thaw processing, incorporating CaCl_2_ and LiCl as antifreeze salts to produce composite fabrics with balanced mechanical flexibility and ionic conductivity. Suppression of ice crystal formation during cooling yielded a uniform amorphous PVA network, in which “free” hydroxyl groups formed strong hydrogen bonds with carbonyl-rich polyester fibers, creating a robust interface. Additional physical interlocking arose from the blend of polyester and polyurethane fibers: polyurethane’s low modulus and high ductility mitigated stiffness mismatch and reduced fiber pull-out under strain. CaCl_2_ and LiCl improved moisture retention and frost resistance, enabling operation in cold environments. The resulting hydrogel–fabric composite showed high sensitivity and stable strain response, suitable for motion and health monitoring. From a crosslinking perspective, this system leverages physical hydrogen bonding and salt-induced coordination rather than extensive covalent crosslinking, prioritizing comfort, reversibility, and low-temperature performance over extreme toughness.

Chen et al. [226] approached hydrogel-based smart textile systems from a circuit-integration perspective, using a printing–cutting–transfer process to embed high-precision copper circuits into fabrics and combining them with hydrogel batteries for safe, low-power operation. A multilayer trace-bundling strategy allowed chip integration without rigid islands, improving wear comfort and mechanical compliance. Backscatter communication minimized power consumption, and hydrogel batteries provided environmentally friendlier, intrinsically safer storage compared with conventional rigid cells. While detailed hydrogel chemistry was not the primary focus, this work underscores the importance of pairing compliant interconnects and power sources with textile substrates for fully integrated smart garments.

Sun et al. [227] developed an all-in-one active pressure-sensing fabric (APPS) that integrates a zinc–air soft-matter battery with a hydrogel-based pressure sensor in a thin (~0.7 mm) textile form factor. A solid neutral hydrogel electrolyte was used both for safety (non-flammable, low leakage) and mechanical stability, supporting an open-circuit voltage of 1.0 V, short-circuit current of 35 mA/cm^2^, and capacity of 2.6 mAh/cm^2^—enough to power microcontrollers and Bluetooth modules. The pressure sensor exhibited high sensitivity (19.6 Ω^−1^·kPa^−1^), a fast response time (9 ms), and stable performance over 6000 cycles, detecting subtle skin pressure and motion when packaged as an adhesive bandage. The hydrogel network relied on covalent crosslinking for structural integrity and dense ionic pathways, with additional physical interactions to maintain adhesion and fatigue resistance. While its energy density and lifetime do not match those of large-format batteries, the integrated, self-powered sensing concept aligns closely with wearable performance targets: mechanical resilience across repeated cycles, rapid response, and safe, skin-compatible operation.

Hydrogels functionalize fabrics by adding compliant, ionically conductive, and often antimicrobial layers that interface intimately with skin and embedded electronics, while textile substrates provide macroscopic strength, breathability, and washability. Across the reported studies, crosslinking strategies—borate–diol coordination in alginate/guar fibers, UV-cured covalent networks reinforced by nanofiber scaffolds in Zn–Mn batteries, freeze–thaw PVA networks hydrogen-bonded to polyester, and multi-mode networks in soft-matter batteries—are central to reconciling competing requirements of softness, adhesion, toughness, and environmental stability. Many fabric-integrated hydrogel systems now meet or exceed typical mechanical and cyclic targets for wearable use (stable operation over thousands of loading cycles, robust performance after washing or deformation), while energy and power densities of hydrogel-based fiber batteries and hybrid devices are approaching those of early-generation flexible batteries and supercapacitors. The hydrogel-based e-textiles and smart fabrics market is projected to grow at a CAGR of roughly 18.2–42.2% to around US$275 billion by 2034 [228,229], driven by demand for wearable health monitoring, ambient intelligence in clothing, and sustainable textile finishes that move away from fluorinated coatings toward water-based and natural polymer strategies. Together, these developments position hydrogel-based smart fabrics as a key platform for next-generation wearable materials that unite comfort, functionality, and environmental stewardship.

### 4.6. Smart Hybrid Devices

Smart hybrid devices integrate multiple functions—typically energy harvesting, energy storage, and sensing—into a single, compact platform, often designed for compatibility with flexible wearable devices and textiles [230,231,232,233,234,235,236,237,238]. Hydrogels play a central role in these systems because their mechanical compliance, ionic conductivity, and tunable chemistry allow a single material to serve multiple purposes, including acting as an electrolyte, dielectric layer, structural support, and, in some cases, a triboelectric or piezoelectric component.

To maintain functionality under demanding conditions such as large strains, impacts, and long-term cycling, crosslinking mechanisms are carefully engineered. Covalent networks provide structural integrity, physical interactions such as hydrogen bonding and polymer entanglement contribute toughness and self-healing, and ionic or coordination bonds offer dynamic reinforcement and strong adhesion. These strategies ensure that smart hybrid devices meet durability and conductivity targets similar to those outlined for wearable batteries and supercapacitors, while enabling multifunctionality in a single, integrated platform.

Li et al. [230] reported an integrated wearable device that combines a solid-state Zn^2+^ battery and a highly sensitive capacitive pressure sensor using a single ionic gel as both electrolyte and dielectric element. In this architecture, the ionic gel—crosslinked via covalent polymerization and reinforced by ion–polymer interactions—simultaneously serves as the zinc battery electrolyte, the sensor’s dielectric layer, and the medium embedding VO_2_ nanoneedle electrodes. The zinc battery module delivered a discharge specific capacity of 28 mAh/g at 0.1 A/g and maintained consistent output even under cyclic pressure and extreme impacts, demonstrating mechanical resilience and electrochemical stability. The capacitive sensor module exhibited ultrahigh sensitivity (23,000 kPa^−1^), rapid response, and accurate detection of fingertip pulses and respiration patterns over 7000 loading/unloading cycles at 40 kPa. Compared with the target values in Table 1, the battery’s specific capacity is modest relative to high-energy, flexible Li-ion cells but acceptable for thin Zn-based storage. At the same time, the sensor’s sensitivity and cycling stability clearly exceed typical benchmarks for flexible pressure sensors, highlighting the value of a shared ionic-gel framework.

Wang et al. [231] developed a wearable self-powered quasi-solid-state (QSS) zinc-ion hybrid supercapacitor (ZIHSC)-type pressure sensor that co-locates energy storage and sensing in a single device. The ZIHSC employed a PEDOT:PSS capacitor-type cathode, a ZnSO_4_–PAM hydrogel electrolyte, and a graphite paper@zinc anode. The hydrogel electrolyte formed a covalently crosslinked PAM network swollen with ZnSO_4_ solution, ensuring ionic conductivity and mechanical robustness under deformation. The ZIHSC achieved a capacitance of 165.2 mF/cm^2^, energy density of 66.3 μWh/cm^2^, power density of 1785 μWh/cm^2^ and 99.3% capacitance retention after 10,000 cycles. As a pressure sensor, it covered a wide detection range (1–110 kPa) with response times below 300 ms. These storage metrics fall within or above supercapacitor-level targets in Table 1 (kW/kg-scale power and 1000–10,000 cycle life), while the sensing performance (broad pressure range, fast response, and long cycling) satisfies practical requirements for wearable pressure monitoring, all enabled by a hydrogel that balances conductivity and toughness.

Liu et al. [232] developed a tree-shaped microneedle platform (GP-eMN) that integrates a flexible TENG with a bioelectronic system for closed-loop therapy and sensing. The base layer is a conductive GelMA hydrogel infused with silver nanowires (AgNWs), providing electronic conduction and mechanical compliance; the top microneedles are formed from an adjustable PVA hydrogel loaded with metformin. Covalent crosslinking in GelMA (photo-initiated) and PVA (physical/chemical crosslinks), together with percolating AgNW networks and hydrogel–substrate adhesion, support mechanical stability and safe skin interfacing. The device harvests biomechanical energy via the TENG to generate electrical pulses that stimulate tissue regeneration, while microneedles deliver drugs and measure glucose, uric acid, and pH at the wound site for real-time monitoring. Although the explicit energy and power densities are not directly compared with Table 1, the architecture leverages hydrogel crosslinking to maintain function under bending and motion while adding sensing and therapeutic capabilities in a single, minimally invasive platform.

Ge et al. [233] designed a poly(vinyl alcohol)/poly(acrylamide)/zinc sulfate (PPZ) hydrogel that simultaneously serves as a triboelectric electrode, current collector, and electrolyte in an integrated self-charging power system. The PPZ hydrogel, formed via covalent crosslinking of PVA and PAAm and ionic incorporation of ZnSO_4_, exhibits a percolating ionic network and robust mechanical matrix. Encapsulated in silicone rubber, the PPZ-based TENG (PPZ-TENG) delivered an open-circuit voltage of 176 V, short-circuit current of 3.56 μA, and power density of 328 mW/m^2^, with stable output after 12,000 cycles and high sensitivity to limb motion. Complementary PPZ-based ZIBs with Zn anodes and MnO_2_ cathodes provided robust, deformation-tolerant storage, enabling the combined system to harvest mechanical energy and store it to power calculators, watches, and headlamps. Mechanically, the hydrogels meet high-strain requirements and show long cycling, while the power density and voltage output are competitive for wearable energy harvesting. Although the ZIBs’ energy density is still below that of advanced flexible Li-ion batteries, the integrated harvesting–storage approach delivers continuous, distributed power at the point of use.

Across these examples, smart hybrid hydrogel-based devices illustrate how judicious crosslinking and composite design can merge energy storage, energy harvesting, and sensing into compact, deformable platforms. Covalent networks ensure structural integrity and defined ion pathways; physical and ionic/coordination crosslinks provide toughness, self-healing, and strong adhesion to soft tissues (e.g., human skin) or substrates; and embedded conductive or piezoelectric fillers deliver the necessary electronic or triboelectric response. As hydrogels continue to be engineered to match or surpass target ranges for conductivity, mechanical durability, and cycle life under realistic wearable conditions, such hybrid systems are poised to become key building blocks for self-powered, intelligent sensing platforms in health monitoring, soft robotics, and ubiquitous IoT applications.

### 4.7. Artificial Intelligence in Hydrogel-Based Wearables

Artificial intelligence (AI) and machine learning (ML) are increasingly being integrated into hydrogel-based wearables to optimize material design, interpret complex sensor outputs, and enable adaptive, “intelligent” behaviour. In these systems, hydrogels provide soft, conformal, and often self-powered sensing platforms, while AI and ML models process high-dimensional, noisy data to enhance accuracy, robustness, and personalization.

Stable and repeatable signal generation is essential for effective AI-driven interpretation, and this depends on the underlying crosslinking design of the hydrogel. Covalent networks ensure structural integrity, physical interactions such as hydrogen bonding and π–π stacking contribute toughness and self-healing, and ionic or coordination bonds enable dynamic responsiveness. Together, these mechanisms create reliable sensing interfaces that AI models can learn from and interpret consistently over extended periods, paving the way for smarter, more responsive wearable technologies.

Boztepe et al. [239] used AI to model the deswelling behaviour of temperature- and pH-responsive poly(N-isopropylacrylamide-co-acrylic acid) interpenetrating polymer network (poly(NIPAAm-co-AAc) IPN) hydrogels. These IPNs combine at least two crosslinked networks—typically one NIPAAm-rich and one AAc-rich—linked by covalent crosslinkers, with additional hydrogen bonding and ionic interactions between amide and carboxyl groups. This multi-network structure yields nonlinear, coupled responses to temperature and pH, making analytical modelling difficult. Artificial neural network (ANN) and least-squares support vector machine (LS-SVM) models were trained on experimental deswelling data and showed high correlation with measured behaviour, demonstrating strong predictive capability. Such models help map composition and crosslinking density to swelling kinetics, guiding hydrogel design for controlled actuation or drug release in wearables, even though they do not directly address full device-level performance targets like cycle life or power consumption.

Elnemr et al. [240] developed a hydrogel-based smart wearable for environmental monitoring (temperature and UV exposure) using digitally processed 3D printing. The hydrogel formulation combined HEMA, PEGDA, and TPO, with PEGDA providing covalent crosslinking via photopolymerization and HEMA contributing hydrophilicity and mechanical softness. This network incorporated photochromic or thermochromic moieties, enabling dynamic colour changes in response to thermal or UV stimuli. An AI model then translated RGB colourimetric changes into quantitative temperature readings with ~90% accuracy across a range of conditions. Here, the crosslinked network ensures stable optical and mechanical properties, while AI compensates for nonlinearity and environmental noise, enabling a non-invasive, robust environmental sensor without complex electronics at the sensing site.

Li et al. [241] developed an MXene-based organohydrogel (M-OH) sensor for health monitoring and machine-learning-assisted object recognition. The hydrogel comprises a dual-network PVA/PAM matrix, MXene flakes, and a lithium salt; covalent crosslinking of PAM, physical crosslinking (hydrogen bonds, crystallite domains) in PVA, and strong interfacial interactions between MXene and polymer chains produce a robust, conductive network. This structure enables 2000% stretchability, high conductivity (4.5 S/m), durable environmental stability (>6 months), and cold tolerance to −24 °C. An 8 × 8 pixelated M-OH sensor array coupled with a deep neural network (DNN) for pressure-mapping image recognition was able to identify different objects with 97.54% accuracy. In terms of performance targets, the mechanical and conductive properties match or exceed those expected for high-performance wearable strain/pressure sensors, and AI elevates the system from simple signal acquisition to complex pattern recognition for human–machine interfaces and intelligent prosthetics.

Li et al. [242] demonstrated an AI-enhanced, self-powered hydrogel system for material identification based on thermal properties. They fabricated a dual-network hydrogel containing a thermogalvanic system—using [Fe(CN)_6_]^3−/4−^ as a redox couple—and a mechanically robust polymer network optimized with lithium magnesium silicate, guanidinium (Gdm^+^), and lithium bromide. Covalent crosslinks formed the structural network, while ionic associations and the thermogalvanic redox species created interconnected ion-conductive pathways and thermally responsive behaviour. Temperature gradients between the hydrogel and contacting materials generated electrical signals without external power. A machine learning model was trained on these thermogalvanic signatures to classify materials based on their thermal properties, achieving accurate identification. This work exemplifies how crosslinked thermogalvanic hydrogels combined with ML can enable autonomous material recognition, potentially informing adaptive grip control in robotics or context-aware wearables.

Song et al. [243] fabricated a conductive hydrogel-based electronic skin (e-skin) for sign language recognition in complex environments. The hydrogel network used acrylic acid (AA) as a monomer, aluminum ions (Al^3+^) and bayberry tannin as crosslinkers, PVA as a flexible backbone, and ethylene glycol as a cryoprotectant. AA provided abundant carboxyl groups for ionic and hydrogen bonding, bayberry tannin introduced multiple phenolic groups for strong hydrogen bonds and metal coordination with Al^3+^, and PVA contributed additional physical crosslinks and mechanical softness. Together, these covalent, coordination, and hydrogen-bonded interactions produced a network with fast self-healing, strong adhesion, excellent anti-freezing properties, and moisture retention. Mounted in a smart glove, this e-skin captured complex hand motions, while a deep learning model extracted motion features for sign language recognition with 93.5% accuracy. The hydrogel’s crosslinking architecture ensured stable, low-noise signals under repeated finger flexion, while AI handled temporal and spatial complexity, bringing system-level performance close to practical thresholds for assistive communication devices.

More broadly, hydrogel-based wearables are being paired with AI-driven analytics across applications such as sweat biosensing, motion tracking, and multimodal health monitoring. For example, bilayer PVA composite patches functionalized with colourimetric reagents and enzymes can quantify pH and glucose in sweat, while ML and deep learning models on smartphones achieve near-clinical accuracy (R^2^ ≈ 0.99) for biomarker estimation from images. In parallel, AI frameworks—CNNs, RNNs/LSTMs, Transformers, and multimodal fusion architectures—are increasingly used to handle sequential, high-dimensional data streams from hydrogel sensors, enhancing robustness against optical, thermal, and mechanical noise [244,245]. In all these cases, robust crosslinking strategies (interpenetrating networks, dual/tri-network systems, dynamic covalent/coordination bonds) are crucial for signal stability over thousands of cycles and across environmental variations, providing the reliable physical layer that AI requires.

Together, these studies show that AI and ML do not replace careful hydrogel design; rather, they complement it. Crosslinked hydrogel networks provide mechanically and chemically stable platforms with rich, multidimensional outputs, while AI models extract patterns, compensate for nonlinearity and noise, and enable complex tasks such as object recognition, material identification, sign language translation, and personalized health analytics. As hydrogel chemistries and crosslinking schemes continue to be refined to meet mechanical and electrochemical targets for wearables, and as AI techniques advance in interpretability and efficiency, the integration of intelligent algorithms and soft hydrogel-based devices is expected to accelerate the development of next-generation, adaptive, and user-centric wearable systems.

**Table 2:** Representative studies highlighting the application of hydrogels for intelligent wearables.

Smart Hydrogel-Based Wearables	Application	Hydrogel Role	Key Performance	Relation to Target/ Requirements for Intelligent Wearables	Ref.
PVA/LiCl–MXene SC	All-round SC for cold environments	Electrolyte + electrode interlayer	113.13 mF/cm^2^; ~95% retention under deformation; anti-freezing to −40 °C	Excellent mechanical and temperature robustness; energy density moderate but sufficient for short bursts in textiles and patches	Yin et al. [125]
NaCl/SA/PAM/PNIPAM/CaCO_3_ sensor	Wide-temperature strain and throat/vocalization sensor	Sensing hydrogel	2.75 S/m; elongation 950%, fracture >2000%; GF 8.76; self-healing ~40%	High strain, high GF, wide T range; suitable for sophisticated HMI (gesture, speech, Morse code)	Wang et al. [135]
HE-PAM Zn/MnO_2_ battery	Flexible aqueous Zn battery for wearables	Electrolyte	0.0225 S/cm; 80.05% capacity retention after 2000 cycles at 10 A/g; 0.5–2.0 V window	Meets ionic conductivity and cycle-life targets for flexible Zn cells; energy density below Li-ion targets but adequate for low–mid power wearables	Shen et al. [178]
Bound-water hydrogel K-ion battery	Aqueous K-ion battery	Electrolyte	~1.9 V; >3000 cycles stable	Long cycle life exceeds >1000-cycle target; aqueous, safer chemistry; still lower energy density than flexible Li-ion	Li et al. [179]
S-PAM FZAB	Flexible Zn–air battery integrated in wearables	Electrolyte + mechanical support	113 mS/cm; ~1.3 V; 30 mW/cm^2^; 694 mAh/g; >40 h operation	Power density and capacity strong for Zn–air; good bending tolerance; suitable for patch/garment power modules	Dai et al. [181]
TNPE SC	Solid-state SC for low-T operation	Electrolyte	17 Wh/kg; 671 W/kg; ≈100% capacitance retention after 10,000 cycles at 2.25 V; −30 °C operation	Cycle life and low-T performance exceed typical targets; moderate energy density appropriate for high-power wearable nodes	Je et al. [194]
Cellulose/Zn^2+^/Ca^2+^–PVA/borax DN TENG	TENG electrode and self-powered strain/tactile sensor	Soft ionic conductor and structural network	VOC 176 V (with TENG), ISC 3.56 μA; power 328 mW/m^2^; stable >12,000 cycles; strong adhesion, recyclability	Meets mechanical and cycling requirements for textile TENGs and self-powered touch sensors	Wang et al. [213]
BaTiO_3_–hydrogel PTENG	Piezo-triboelectric hybrid NG	Electrode and active layer	VOC 222 V; ISC 5.35 μA; 2.14 MPa strength; 876% strain	Suitable for powering small electronics and as self-powered strain/tactile sensor in wearable/biomedical settings	Hou et al. [215]
B–SZA/GG fabric fibers	Hydrogel–fiber fabrics for motion sensing and antibacterial wear	Fiber substrate with ionic conduction	40.9 MPa strength; 233% elongation; −23.1 °C freezing point; antibacterial	High mechanical strength and antifreeze properties ideal for hydrogel-functionalized garments	Guo et al. [222]
AA/Al^3+^/tannin/PVA/EG e-skin glove	Sign language recognition e-skin	Conductive, adhesive e-skin	Anti-freezing, self-healing, strong adhesion; 93.5% DL recognition accuracy	Satisfies wearability and robustness; AI adds high-level communication capability in complex environments	Song et al. [243]

**Table 3:** Representative hydrogel-based wearable devices and textiles by material category and crosslinking design.

Smart Hydrogel-Based Wearables	Composition	Source/Sustainability	Crosslinking Mechanism(s)	Key Hydrogel Properties Relevant to Wearables	Ref.
PVA/LiCl antifreeze	PVA hydrogel with LiCl; MXene/CMC electrodes	Synthetic PVA; natural CMC	Physical PVA freeze–thaw network; Li^+^–OH coordination; MXene–CMC H-bonding	Soft, self-adhesive, self-healing; anti-freezing to −40 °C; specific capacitance 113.13 mF/cm^2^; ~95% retention under deformation	Yin et al. [125]
HE-PAM, Zn/MnO_2_ battery	Highly entangled PAM hydrogel; HE-PAM vs. conventional C-PAM	Synthetic acrylamide; water-based	Covalent crosslinking (MBA/APS), enhanced chain entanglement, continuous free-water network	Ionic conductivity 0.0225 S/cm; modulus 166 kPa; stable 6 months; 80.05% capacity retention after 2000 cycles at 10 A/g; wide window (0.5–2.0 V)	Shen et al. [178]
Bound-water supramolecular hydrogel, K-ion battery	PVA + glucose + PSBMA + AM + Alg + laponite	Partly bio-derived (glucose, alginate)	Covalent (radical polymerization), strong hydrogen bonding, zwitterionic and clay-based physical interactions tuning water states	High bound water (>0.4 mg/mg), low free water; high voltage (~1.9 V); >3000 stable cycles; improved low-T and interfacial stability	Li et al. [179]
S-PAM starch-reinforced, Zn–air	Starch-reinforced PAM (S-PAM) electrolyte	Natural starch + synthetic PAM	Covalent PAM network; starch–polymer hydrogen bonding and entanglement	Conductivity 113 mS/cm; discharge voltage ~1.3 V; power density 30 mW/cm^2^; 694 mAh/g; >40 h stable under bending/impact	Dai et al. [181]
CMC/PAM double-network	Carboxymethyl cellulose (CMC) + PAM DN hydrogel	Natural CMC + synthetic PAM	First covalent (PAM), second H-bond/ionic network (CMC–PAM); DN design	Tensile strain 2700%; conductivity 0.0637 S/cm; areal capacitance 170.23 μAh/cm^2^; 73.14% retention after 100 cycles	Li et al. [182]
GCZ-x CM-chitosan/gelatin	Carboxymethyl chitosan + gelatin + Zn^2+^ salts	Natural chitosan + gelatin	Multiple noncovalent interactions (H-bonding, hydrophobic interactions), Hofmeister effects, ionic coordination	Recyclability >80%; stimuli-responsive (T, pH); dendrite-free Zn; 2200 h cycling at 0.1 A/g	Ji et al. [183]
PAA–VSNP hydrogel, Na SHSC	PAA + vinyl-functionalized silica NPs + Na^+^ salt	Synthetic	Covalent PAA network; SiO_2_ NP covalent/physical crosslinking; H-bonding	Stretchability ~1123%; conductivity 20.23 mS/cm; Ea 0.106 eV; SHSC energy 86.95 Wh/kg; power 19.94 kW/kg; >4000 cycles	Kim et al. [196]
BC/PAM hydrogel (BC-reinforced electrolyte)	Bacterial cellulose (BC) nanofibers + PAM	Bacterial cellulose + synthetic PAM	Physical H-bonding between BC –OH and PAM; physical network reinforcement	Conductivity 125 mS/cm; elongation ~1300%; tensile strength 330 kPa; areal capacitance 564 mF/cm^2^; stable under bending	Li et al. [198]
CMC-lignin (lignin-based electrode hydrogel)	N-doped carbon dots + graphene hydrogel (corncob lignin source)	Biomass-derived lignin + graphene	Physical gelation via π–π stacking, electrostatic interactions	387 F/g at 1 A/g; 92.3% retention after 5000 cycles; energy 25.6 Wh/kg at 243 W/kg; stable under bending	Cui et al. [199]
BaTiO_3_-doped alginate/AA/AM/Fe^3+^ hydrogel	Oxidized SA + AA + AM + BaTiO_3_ + FeCl_3_	SA partially bio-based	Dynamic Schiff base, Fe^3+^–carboxylate coordination, H-bonding	Tensile stress 2.14 MPa; strain 876%; conductivity 0.14 S/m; VOC 222 V; ISC 5.35 μA (PTENG)	Hou et al. [215]

Table 4 indicates the role of hydrogels as a cornerstone for advancing wearable technologies, offering unprecedented flexibility, biocompatibility, and adaptive functionality for next-generation smart textiles and on-skin devices. The examples in Table 4 illustrate a rapid evolution: from water-based natural polymer hydrogel coatings on textile substrates to integrated systems for biosensing and energy harvesting. Innovators are aligning wearable design with both environmental priorities and enhanced device efficiency. Attention to eco-friendly composition, such as biopolymer selection and minimized use of hazardous agents, reflects growing market and regulatory demands for greener solutions in wearable technology.

**Table 4:** Representative patented hydrogels for wearable devices and textiles.

Publication	Assignee	Hydrogel Composition/ Functionalization	Integration Method	Wearable Function	Eco-Evidence
US 2021/0137402 A1 [246]	Arizona Board of Regents (ASU)	Hydrogel providing ionic interface to Ag/AgCl; iCVD surface treatments (pHEA/pFDA) for stability	Hydrogel coated/deposited on conductive threads stitched to a fabric	Sensor/electrode (EOG/EEG/EMG/ECG)	Inferred: On-skin biocompatible hydrogel interface; hydrogel processing compatible with textiles; no explicit green claim.
US 11,272,868 B2 [247]	The Regents of the University of California	Hydrogel reservoir and hydrogel salt bridge (e.g., agarose) with reference electrolyte	Thin, flexible on-skin package; printed Ag/AgCl; bandage-like form	Sensor (sweat ions)	Direct: Natural polymer agarose hydrogel; water-based electrolyte layers.
US 2023/0397870 A1 [248]	The General Hospital Corporation	PVA-based dynamic covalent hydrogel (boronic ester) with conductive fillers (CNTs, PEDOT:PSS, MXene; optional chitosan)	Cast/moulded hydrogel electrodes in soft encapsulation for skin contact	Sensor/electrode	Inferred: Aqueous-processable PVA/chitosan; focus on skin safety and low impedance rather than explicit sustainability.
US 2017/0325724 A1 → US 10,722,160 B2 [249]	The Regents of the University of California	Hydrogel transport layer to convey interstitial fluid analytes to electrodes	Layered flexible patch over working/reference electrodes	Sensor/biosensor	Inferred: Hydrogel used for biocompatible transport; centers on performance, not environmental claims.
US 11,476,780 B2 [250]	City University of Hong Kong	Soft interfaces compatible with flexible straps; hydrogel compatible as ionic layer/encapsulant (conceptual)	Harvester integrated into wrist strap and housing	Harvester/hybrid	Inferred: Hydrogel compatibility noted conceptually; eco aspects not claimed.
US 2024/0182650 A1 [21]	Yee Tung Garment Company Limited	Carboxymethyl cellulose hydrogel crosslinked (e.g., fumaric acid), cavities for agents (e.g., ZnO, xylitol)	Hydrogel coating applied to fabric substrates for garments	Smart fabric (comfort/self-cooling fabrics)	Direct: Natural polymer (CMC), water-based chemistry; textile coating focus suggests reduced solvent reliance.

## 5. Challenges and Proposed Solutions

Hydrogels offer compelling advantages for intelligent wearable devices and textiles, including skin-like mechanics, biocompatibility, ionic/electronic conductivity, and potential sustainability. However, current hydrogel systems still fall short of several key target values for high-performance devices, particularly in energy density (vs. flexible Li-ion batteries), long-term environmental stability (months–years), and reproducibility under realistic mechanical and thermal cycling conditions. Conventional single-polymer-network hydrogels often lack sufficient strength, toughness, and fatigue resistance for long-term use, and their strong dependence on water content makes them vulnerable to dehydration, freeze–thaw damage, and humidity- or temperature-induced drift in conductivity and signal response. These issues directly affect the stability and reliability of hydrogel-based batteries, supercapacitors, sensors, nanogenerators, and fabric systems.

Dehydration and environmental instability remain primary bottlenecks. Loss of water degrades ionic conductivity, alters modulus, and destabilizes sensing baselines, leading to noisy or drifting signals in strain and pressure sensors and device interfaces. This is especially critical for applications requiring long-term continuous monitoring (e.g., cardiovascular patches, smart garments), where even small shifts in impedance or capacitance can reduce accuracy. For energy devices, hydrogel electrolytes should maintain ionic conductivity in the 0.001–0.1 S/cm range while resisting drying, crystallization, and phase separation. At the same time, many high-performance formulations rely on organic cosolvents, salts, and nanofillers that can complicate biocompatibility and recyclability. Integrating hydrogels with electrodes, circuits, and textiles adds further complexity: weak hydrogel–substrate interfaces lead to delamination, cracking, or loss of adhesion under strain, while mismatched stiffness can cause discomfort, localized stress, and premature failure.

Scalability and manufacturability are equally challenging. Current studies employ sophisticated crosslinking strategies (double/triple networks; covalent–physical–ionic hybrids), careful control of water states (bound vs. free water), optimized filler dispersion (MXene, CNTs, BaTiO_3_, CCTO, AgNWs), and multiscale architectures (microneedles, fibers, multilayer composites). Reproducing such structures at an industrial scale with consistent performance is non-trivial. Batch-to-batch variations in polymerization conditions, nanofiller loading, or solvent composition can lead to ±10–30% variability in mechanical or electrical properties, which is problematic for scaling up or for safety-critical applications. Standard textile processes (weaving, knitting, dyeing, washing) impose additional constraints; hydrogels must survive mechanical agitation, detergents, drying, and ironing while retaining conductivity and adhesion.

Addressing these challenges requires coordinated strategies at the material, device, and system levels. At the material level, multi-network and composite designs should continue to be refined to simultaneously achieve: (i) toughening networks (e.g., DN/TN architectures with sacrificial bonds, ion coordination, nanofiber reinforcement) that match or exceed mechanical targets (tensile strength >1 MPa, elongation >400%, toughness in the MJ/m^3^ range); (ii) conductive networks (ionic and/or electronic) delivering stable conductivity in the 0.001–1 S/m range across −20 to 60 °C; and (iii) controlled water states (lean-water, bound-water-dominant formulations) that minimize dehydration and freeze–thaw effects. Crosslinking chemistry is a central lever: combining permanent covalent backbones with reversible physical and coordination bonds enables hydrogels to dissipate energy and self-heal while preserving structural integrity, directly addressing fatigue and damage accumulation under cyclic deformation.

Environmental stability can be improved through solvent engineering (glycerol/water, DMSO/water, ionic liquids), antifreeze salts (LiCl, CaCl_2_, NaCl), and encapsulation strategies (thin elastomer shells, barrier coatings) that slow water loss without sacrificing breathability. For wearable devices and textiles, thin, breathable encapsulation layers and optimized thicknesses can balance protection against dehydration with skin comfort and infection risk. At the integration level, interfacial design must address mechanical and chemical bonding between hydrogels and textiles, as well as between hydrogels and electrodes and circuits. Approaches include surface functionalization of fibers and electrodes, interpenetrating interfacial networks, and graded-stiffness layers (e.g., hydrogel–PU–polyester stacks) to reduce stress concentrations. These strategies are already visible in smart fabric (Section 4.5) and sensor (Section 4.3) sections and should be systematically extended to batteries, supercapacitors, and nanogenerators.

Reproducibility and manufacturing scalability require standardization and process-compatible chemistries. Photocuring, controlled radical polymerization, inkjet or extrusion printing, and roll-to-roll coating/lamination can provide better control over crosslink density, patterning, and layer thickness than batch casting or hand mixing. For textiles, adapting to established processes (dip-coating, screen printing, yarn coating, in-fiber spinning) while preserving the designed microstructures is crucial. Combining in-line sensing and AI/ML-based process control (e.g., using low-cost sensors to track curing, moisture content, or conductivity during production) can help maintain batch consistency.

At the system level, AI and machine learning can mitigate some material limitations by compensating for drift, nonlinearity, and inter-individual variability. As seen in Section 4.7, AI can correct for environmental influences (e.g., temperature, pH) on signal outputs, enhance pattern recognition (e.g., sign language, object identification, gait analysis), and adapt device behaviour to user-specific patterns. Importantly, this should not be treated as a substitute for material robustness; rather, AI can extend the useful operating window of well-engineered hydrogels and accelerate design optimization (e.g., mapping composition and crosslinking parameters to target mechanical or electrochemical metrics).

Finally, translation from lab-proof-of-concept to commercial hydrogel-enabled wearables requires close collaboration among polymer chemists, device engineers, textile scientists, clinicians, and industry partners. Academia contributes fundamental understanding of hydrogel mechanics, conduction, and biocompatibility, as well as new crosslinking and composite strategies. Industry brings expertise in scale-up, quality control, regulatory pathways, and user-centered product design. Public–private consortia focused on standardized test protocols (e.g., mechanical fatigue under sweat and temperature cycling, wash durability, skin irritation and sensitization), reference materials, and open datasets for AI/ML training would significantly de-risk commercialization. These efforts, combined with advances in sustainable feedstocks (cellulose, chitosan, lignin, gelatin) and recyclable device architectures, can accelerate the deployment of hydrogel-based intelligent wearables and smart textiles that meet performance, safety, and environmental targets.

## 6. Conclusions, Outlook and Future Perspectives

This review highlights that hydrogel-based intelligent wearables have rapidly evolved from proof-of-concept devices and textiles to multifunctional platforms that combine sensing, energy storage, energy harvesting, communication, and computation. Conductive and composite hydrogels now underpin flexible Zn-based batteries and Zn–air cells, high-power supercapacitors, strain and pressure sensors with high gauge factors, triboelectric and piezoelectric nanogenerators, smart fabrics, and hybrid systems that integrate multiple functions in a single architecture. Many of these devices already meet or exceed key metrics for wearable operation—such as strain tolerance (hundreds to thousands of percent), power density in the kW/kg range, cycle lifetimes of 1000–10,000 cycles, and stable performance at sub-zero temperatures—while maintaining comfort and biocompatibility.

Future research should focus on closing the remaining gaps between hydrogel-based systems and the best-in-class rigid or semi-rigid technologies, particularly in energy density and long-term environmental stability. For energy storage, a key challenge is to raise the Wh/kg and Wh/L of hydrogel-based Zn, Na, and hybrid systems toward or beyond the lower end of flexible Li-ion performance, without sacrificing safety or mechanical compliance. This will likely require more aggressive integration of high-capacity electrode chemistries with lean-water or quasi-solid hydrogel electrolytes that maintain high ionic conductivity while suppressing parasitic reactions and dendrite formation. For supercapacitors, continued optimization of conductive polymer and MXene-based hydrogel networks could yield devices that approach battery-level energy densities while preserving high power and cycling stability.

For future hydrogel-based wearables, multifunctionality will be increasingly important. Wearables and textiles that simultaneously sense strain, pressure, temperature, and biochemical markers; harvest energy from motion or heat; and provide localized therapy (e.g., drug release, electrical stimulation) are already emerging in the revised sections (e.g., microneedle platforms, self-powered fabrics, integrated HTS garments). It will likely integrate additional modalities such as optical sensing, ultrasound, or chemical imaging, demanding hydrogels that can host multiple transduction mechanisms without cross-interference. This will push material design toward hierarchical, spatially programmed hydrogels—where different regions or layers provide distinct mechanical, electrical, and biochemical functions.

Another frontier is the deep integration of hydrogels with AI, IoT, and edge computing. As shown in Section 4.7, AI models can transform hydrogel sensor outputs into actionable insights with high accuracy, enabling complex tasks such as sign language translation, object identification, and material classification [25]. Future directions include: (i) on-device learning using low-power neuromorphic hardware embedded in textiles or patches; (ii) federated learning approaches that personalize models to individual users while preserving privacy; and (iii) closed-loop systems where hydrogels not only sense but also actuate (e.g., drug release, stiffness modulation) in response to AI-driven decisions. Achieving this will require careful co-design of materials, electronics, and algorithms to ensure that hydrogel signals remain stable enough for robust model training and inference.

Sustainability and circularity will become non-negotiable design constraints [2,251]. Many of the systems reviewed already make use of natural polymers (cellulose, starch, alginate, chitosan, gelatin, hyaluronic acid) and biomass-derived fillers (lignin-based carbons) and adopt water-based processing where possible. Future work should extend this by: (i) designing hydrogels with end-of-life in mind (e.g., controlled biodegradation, disassembly, or recycling of functional components); (ii) minimizing reliance on scarce or toxic metals and solvents; and (iii) assessing full life-cycle impacts of hydrogel-enabled wearables versus conventional devices. Standardized eco-design frameworks and regulatory guidance tailored to soft, bio-integrated wearables will help guide material choices and device architectures toward lower environmental footprints and alignments with societal expectations.

Translation into clinical and consumer products will hinge on systematic validation in realistic environments. For health applications, this means long-term studies across diverse populations that account for sweat, movement, the skin microbiome, and hygiene behaviours; rigorous evaluation of skin compatibility, infection risk, and data security; and integration into healthcare workflows. For consumer and industrial wearables, robustness to washing, UV exposure, chemical contamination, and mechanical abuse will be critical. Real-world deployments of smart garments and patches that leverage hydrogel-based power and sensing will generate invaluable data to refine both materials and AI models.

In summary, hydrogel-based intelligent wearables and smart textiles have moved from conceptual promise to a robust, rapidly maturing field. The combination of advanced crosslinking designs, composite architectures, and AI-assisted signal interpretation is enabling devices that are softer, more adaptive, and more functionally rich than traditional rigid electronics. Continued progress will depend on aligning material innovations with system-level requirements and societal priorities—performance, comfort, safety, privacy, and sustainability. With coordinated interdisciplinary efforts and thoughtful integration of AI and IoT, hydrogel-enabled platforms are well-positioned to underpin the next generation of personalized, autonomous, and environmentally responsible wearable technologies.

## Data Availability

No new data were created or analyzed in this study.
